# Cobalt-Doped Carbon Quantum Dots Work Synergistically with Weak Acetic Acid to Eliminate Antimicrobial-Resistant Bacterial Infections

**DOI:** 10.1021/acsnano.5c03108

**Published:** 2025-09-08

**Authors:** Adam Truskewycz, Benedict Choi, Line Pedersen, Jianhua Han, Melanie MacGregor, Nils Halberg

**Affiliations:** † Department of Biomedicine, 1658 University of Bergen, Bergen 5009, Norway; ‡ Flinders Institute for Nanoscale Science and Technology, College of Science and Engineering, 1065 Flinders University, Adelaide, South Australia 5042, Australia; § Department of Biochemistry and Biophysics, University of California, San Francisco, California 94143, United States; ∥ Department of Urology, University of California, San Francisco, California 94143, United States; ⊥ Helen Diller Family Comprehensive Cancer Center, University of California, San Francisco, California 94143, United States; # Bakar Computational Health Sciences Institute, University of California, San Francisco, California 94143, United States; ¶ Cancer Research Program, 56362 QIMR Berghofer Medical Research Institute, Brisbane 4006, Australia

**Keywords:** antimicrobial resistant, nanoparticles, carbon quantum dot, cobalt, antimicrobial, acetic acid

## Abstract

When pathogenic bacteria colonize a wound, they can create an alkaline ecological niche that selects for their survival by creating an inflammatory environment restricting healthy wound healing to proceed. To aid healing, wound acidification has been exploited to disrupt this process and stimulate fibroblast growth, increase wound oxygen concentrations, minimize proteolytic activity, and restimulate the host immune system. Within this study, we have developed cobalt-doped carbon quantum dot nanoparticles that work together with mild acetic acid, creating a potent synergistic antimicrobial therapy. The acidic environment alters the osmotic balance of microorganisms, forcing them to swell and speed up the internalization of the ultrasmall particles. The particles hyperpolarize the bacterial membranes and generate damaging peroxidase species, resulting in cellular lysis. In mice, cobalt-doped carbon quantum dots remove MRSA infection while allowing wounds to heal at rates equivalent to that of uninfected wounds. This work demonstrates how synergistic antimicrobial treatment strategies can be successfully used to combat antimicrobial-resistant infections.

## Graphical abstract

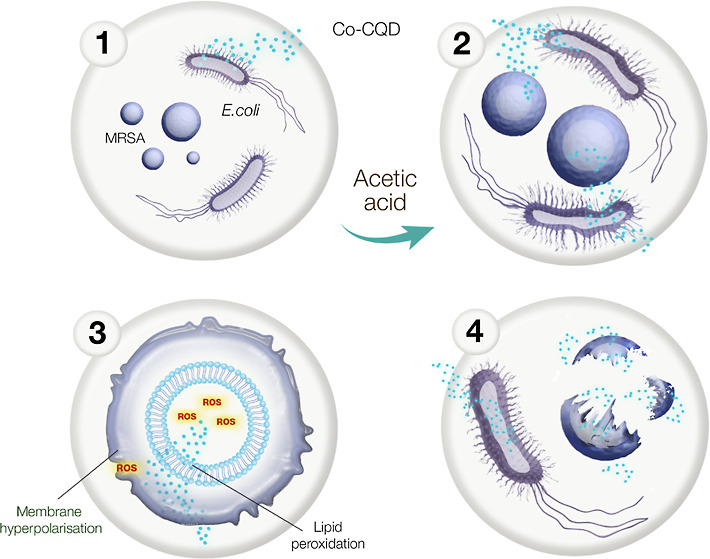

There are currently >4.5 million deaths associated with bacterial antimicrobial resistance (AMR) globally, arising from either a direct infectious disease or through additive physiological stress experienced by those already suffering from pre-existing conditions (i.e., cancer, organ transplantation, HIV, liver and kidney disease, diabetes etc.). Antimicrobial resistance is an increasingly serious threat with too few drugs in the developmental pipeline (WHO)^1^ adept to combat the most adaptive and resilient species. Most new drug candidates are variations of pre-existing commercial chemical antibiotics; however, there is a pressing need for innovative treatments. There are currently only 2 drugs in the pipeline fulfilling at least one of the WHO’s four innovation criteria, which include: drugs with a new chemical class, new microbial targets, new mechanism of action, and/or free from cross antimicrobial resistance.^2^

Ultrasmall nanoparticles hold great promise as antimicrobial agents having the potential to address several of the WHO antimicrobial innovation criteria as they possess properties of both molecular/chemical and physical states.^3^ Their structure separates them from traditional chemical drugs and provides a platform for multifunctional activity, which can further challenge resistance. Carbon quantum dot nanoparticles make up a class of ultrasmall nanoparticles. Unlike other rigid carbon nanoparticles (carbon nanotubes, graphene etc.), they possess high hydrophilicity and monodispersity, making them less likely to be bioaccumulated and have shown a high degree of biocompatibility in numerous studies.^4–6^ Their carbon core contains varying organic chemical structures providing them with abundant functionalization opportunities (i.e., metal dopants and/or organic ligands), to instill them with multiple functional characteristics.^7^

Despite their potential, their ability to remove infection *in vivo* and restore pathogen-infected wounds remains unexplored.

Healthy wound healing proceeds through four main phases, namely, hemostasis, inflammation, proliferation, and remodeling. During these stages, the environments pH fluctuates with (i) an acidic inflammation stage which reduces microbial colonization and promotes vascular regeneration, (ii) a marginally alkaline granulation stage (pH 7.0–7.5) to promote cell proliferation and skin remodeling, followed by a (iii) reacidification of the environment to restore the skin’s acid mantle.^8^ However, infected wounds often remain fixed in the inflammatory state through the action of persistent pathogenic microorganisms. Preventing these microbial infections from forming is key to ensuring healthy wound healing processes.^9^

Pathogenic microorganisms create an environmental niche which is beneficial for their survival while inhibiting the host’s immune system from functioning to control their presence.^10^ An alkaline pH (pH 7.0–9.0),^11^ reduced wound oxygen content,^12^ and increased temperature are frequently associated with seriously infected wounds.^13^ Healing wounds undergo natural acidification, producing lactic acid, which minimizes wound infection and supports vascular migration, DNA replication, wound oxygenation, collagen formation immune activity, and growth of blood vessels.^14^ Therefore, wound acidification has been suggested as a viable approach for reducing infection.^15^ Acetic acid is considered a mild acid, and at diluted concentrations, it has been shown to have pro-wound healing properties.^16^ It has also been used against the plague^17^ and has, in recent days, been studied for its antimicrobial activity and it is particularly effective against pathogenic *Pseudomonas aeruginosa*;^18^ however it shows negligible bactericidal activity against many other wound colonizing bacterial species at concentrations^19^ below the threshold for dermal discomfort (3%). Its short residence time in the wound environment (∼1 h)^20^ allows it to temporarily adjust the wound environment without bioaccumulating, allowing healthy wound healing-associated pH fluctuations to continue.

Here we have developed a combination therapy utilizing weak acetic acid (0.06%, pH 5.5) together with novel, ultrasmall, cobalt-doped carbon quantum dot nanoparticles (Co-CQD) as a potent antimicrobial strategy against several pathogenic species including methicillin- and oxacillin-resistant *Staphylococcus aureus* (MRSA), *Escherichia coli*, and *Enterococcus faecalis*. A weak acetic acid environment creates osmotic disruption resulting in cell swelling and more rapid Co-CQD particle uptake. The particles then destroy bacteria cells through the generation of peroxides and hyperpolarizes their membranes. The particles show a high degree of biocompatibility with dermal fibroblasts (dFIB) at concentrations well over the minimum bactericidal concentrations and were able to remove multi-drug-resistant MRSA infections from mouse wounds without reducing wound healing rates. Together, the platform highlights a biocompatible and efficient strategy for combatting bacterial infections *in vitro* and *in vivo* by blending traditional and innovative combination strategies.

## Results and Discussion

### Synthesis and Characterization of Co-CQDs

Carbon quantum dots doped with cobalt were designed as potent antimicrobial nanoparticles due to cobalt’s documented antimicrobial activity^21^ along with carbon quantum dots’ biocompatibility and potential for functional chemical modifications.^22–24^ Transmission electron microscopy (TEM) of the particles demonstrated that they were monodispersed and had sizes ranging between 1.2 and 4.0 nm, with an average size of 2.6 nm (Figures 1A,B and S1A,B). The hydrothermal conditions during CQD synthesis facilitated the integration of hexamine cobalt chloride (HACC)-derived nitrogen and cobalt into the carbon quantum dot core matrix. XPS analysis showed the particles to be comprised of C (64.7 at. %), Co (2.3 at. %), N (3.3 at. %), O (27.5 at. %), Cl (0.3 at. %), and other (1.9 at. %: Figure 1C).

**Figure 1:**
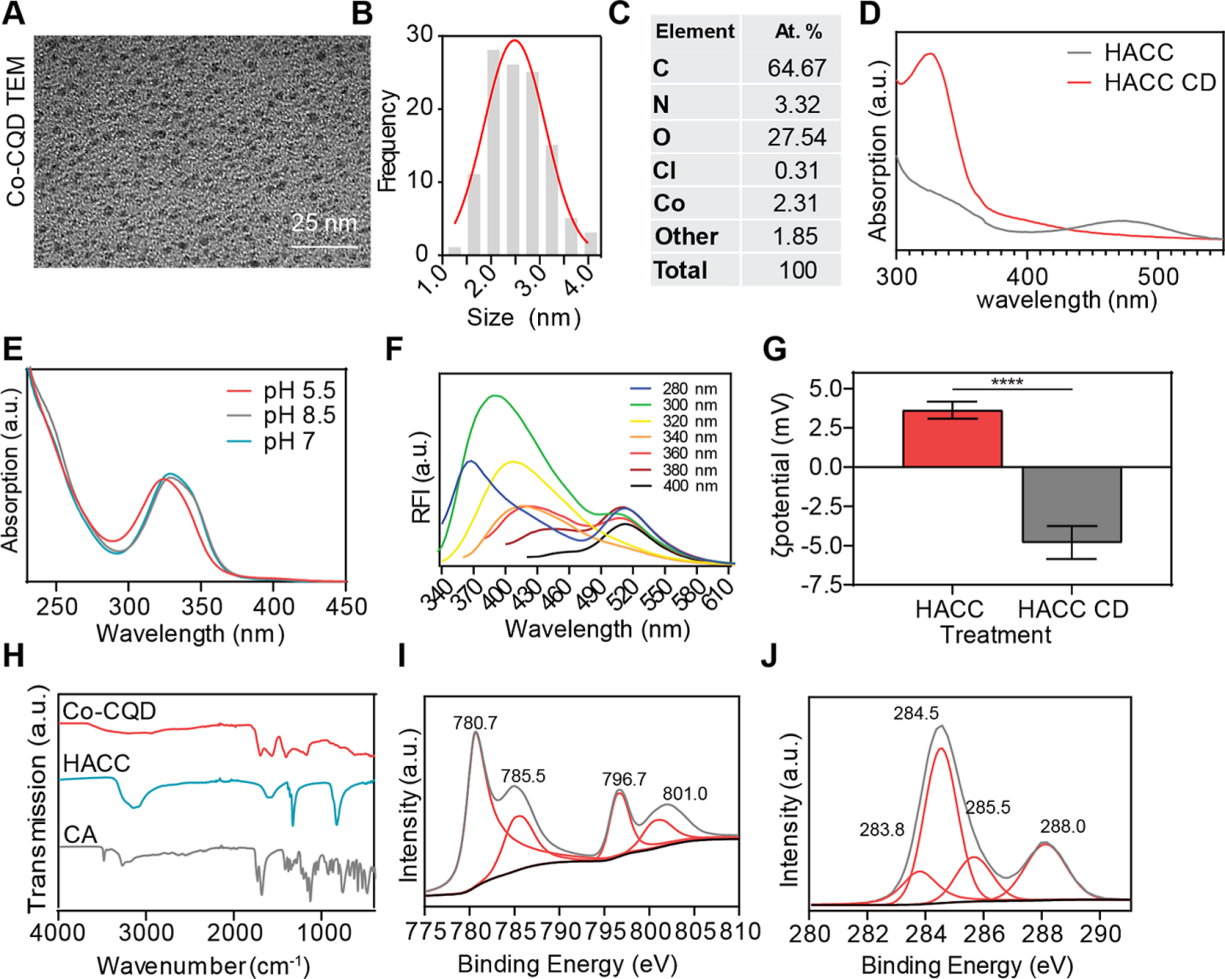
Co-CQD characterization. (A) Representative TEM images of Co-CQD nanoparticles. Scale bar is 25 nm. (B) Histogram of Co-CQD sizes produced through hydrothermal synthesis conditions. (C) X-ray photoelectron spectroscopy (XPS) elemental analysis of Co-CQDs (at. %). (D) Absorbance spectra of Co-CQD and its synthesis precursor hexamine cobalt chloride solution. (E) Absorbance of Co-CQDs in acidic, basic, and alkaline conditions. (F) Excitation and emission fluorescence spectra of Co-CQDs. (G) Zeta potential of hexamine cobalt chloride solution and Co-CQD solution. (H) FTIR spectra of Co-CQDs and the precursors used to synthesize it, namely, citric acid (CA) and hexamine cobalt chloride (HACC). (I) XPS Co 2p peaks from Co-CQDs. (J) XPS C 1s peaks from Co-CQDs. Error bars represent the standard deviation. Statistical significance was determined using the (G) unpaired *t*-test (two tailed). **** signifies *p* < 0.0001.

The absence of the parental HACC compound on the CQDs is observed by differences in their absorbance spectra with the particles possessing a strong peak at 330 nm while losing the HACC characteristic absorbance peak at 435 nm (Figure 1D). The particles show a slight blueshift in absorbance in acidic conditions, indicating breaks in structural conjugation (Figure 1E). The fluorescent particles possess a maximum fluorescence emission at λ_max_ 390 nm when excited with 300 nm light, and incremental red shifting of the peak emissions occurred with increasing excitation wavelengths between 280 and 400 nm (Figure 1F). Variations in zeta potential of the HACC solution and Co-CQDs were shown to switch from a slightly positive to slightly negative charge (3.6 vs −4.8 mV), indicating carboxylic acid functionalization derived from the citric acid precursor (Figure 1G). Nevertheless, these low zeta potential values indicate the particles did not possess strong surface charges. The precursor possessed FTIR peaks at 3250, 1615, 1325, and 823 cm^–1^, which are all representative of N–H bonds. However, these functionalities were absent from the nanoparticles. The nanoparticles displayed carboxylic acid functionalities shown by FTIR spectra peaks at 3300–2500 cm^–1^ (O–H stretch), 1690 cm^–1^ (C=O stretch), 1400 cm^–1^ (O–H bend), and 1227 cm^–1^ (C–O stretch) (Figure 1H).

The Co-CQDs possess a broad Raman D band between 1315 and 1375 cm^–1^, representing a disordered sp^2^ carbonaceous material and a broad Raman G band between 1510 and 1545 cm^–1^ attributable to amorphous carbon (Figure S2A). This was further reinforced through X-ray diffraction analysis, where Co-CQDs showed neither carbon nor cobalt crystalline phases (Figure S2D). The Co 2p XPS spectrum shows two dominant Co^2+^ peaks centered at 780.7 and 796.7 eV for 2p_3/2_ and 2p_1/2_, respectively. Corresponding satellite peaks are centered at 785.5 and 801.0, respectively (Figure 1I). These binding energies coupled with those of the O 1s at 531.1 are characteristic of Co­(OH)_2_ (Figure S2B). The particles possess a nitrogen-containing ring structure as indicated by XPS peaks at 399.1 and 399.8 eV, representative of lactam or cyano nitrogen and pyrrolic nitrogen, respectively (Figure S2C). Carbons C 1s peaks at binding energies of 283.8, 284.5, 285.5, and 288.0 eV (Figure 1J) are assigned to sp^2^ and sp^3^ hybridized carbon, C–OH, and C=O, respectively.

These characterizations reveal that the fluorescent Co-CQDs are monodisperse ultrasmall carbonaceous particles doped with nitrogen and cobalt heteroatoms. The particle structure consists of a disordered carbon-rich core also containing cobalt and nitrogen, with carboxylic acid functionalities present on their surface. The parental synthesis precursor (HACC) is absent from the final nanoparticle product with hydrothermal conditions transforming Co^3+^ into a Co^2+^ oxide.

### Acetic Acid Co-CQDs Exhibit Synergistic Antimicrobial Activity

Methicillin- and oxacillin-resistant *S. aureus* (MRSA, ATCC 43300), *E. coli* (ATCC 25922), and vancomycin-sensitive *E. faecalis* (ATCC 29212) are among the most frequently identified microorganisms in infected wounds^25–27^ and were therefore chosen to investigate the antimicrobial activity of Co-CQD nanoparticles. Acetic acid has been assessed for its antimicrobial activity in preclinical trials and has shown strong antimicrobial activity against the Gram-negative *P. aeruginosa*.^28,29^ It has antibiofilm-forming properties and its undissociated form possesses a neutral charge, allowing it to freely cross bacterial membranes causing cellular acidification and osmotic imbalances. It has been shown to slow the growth of pathogenic bacteria, but its bactericidal activity against these strains is limited. Due to its low dermal toxicity (LD_50_ of 1.06 g/kg in rats), cellular internalization properties, and bacteriostatic nature, acetic acid was selected as a medium for synergistic Co-CQD antimicrobial activity.

The Co-CQD nanoparticles exhibited strong antimicrobial activity toward all strains in aqueous growth cultures, particularly in a weak acetic acid environment (0.06%) (Figure 2A). Acetic acid concentrations of 1–2% (pH ∼ 2.4) have been well tolerated in preclinical trials;^30^ however in this study, we have kept the pH at levels naturally found in healthy healing wounds.^31^ At pH 5.5, all MRSA, *E. coli*, and *E. faecalis* cells were destroyed at a 38, 75, and 75 μg/mL Co-CQD concentration, respectively (minimal bactericidal concentration: MBC) (Figures 2A and S3B). The MIC determined by OD_600_ of antimicrobial overnight culture was 38, 38, and 9 μg/mL, respectively, indicating a bacteriostatic influence on *E. coli* and *E. faecalis*. At pH 7.0, a strong but reduced antimicrobial activity of Co-CQD was observed, indicating the particles exploit the acidic environment for enhanced antimicrobial activity (Figure 2A). At pH 7.0 MRSA, *E. coli*, and *E. faecalis* possessed an MBC of 150, 600, and 300 μg/mL, respectively, and an MIC of 75, 300, and 150 μg/mL, respectively (Figure 2A). An alkaline environment of pH 8.5 further decreased the antimicrobial activity of the particles (Figure S3A).

**Figure 2:**
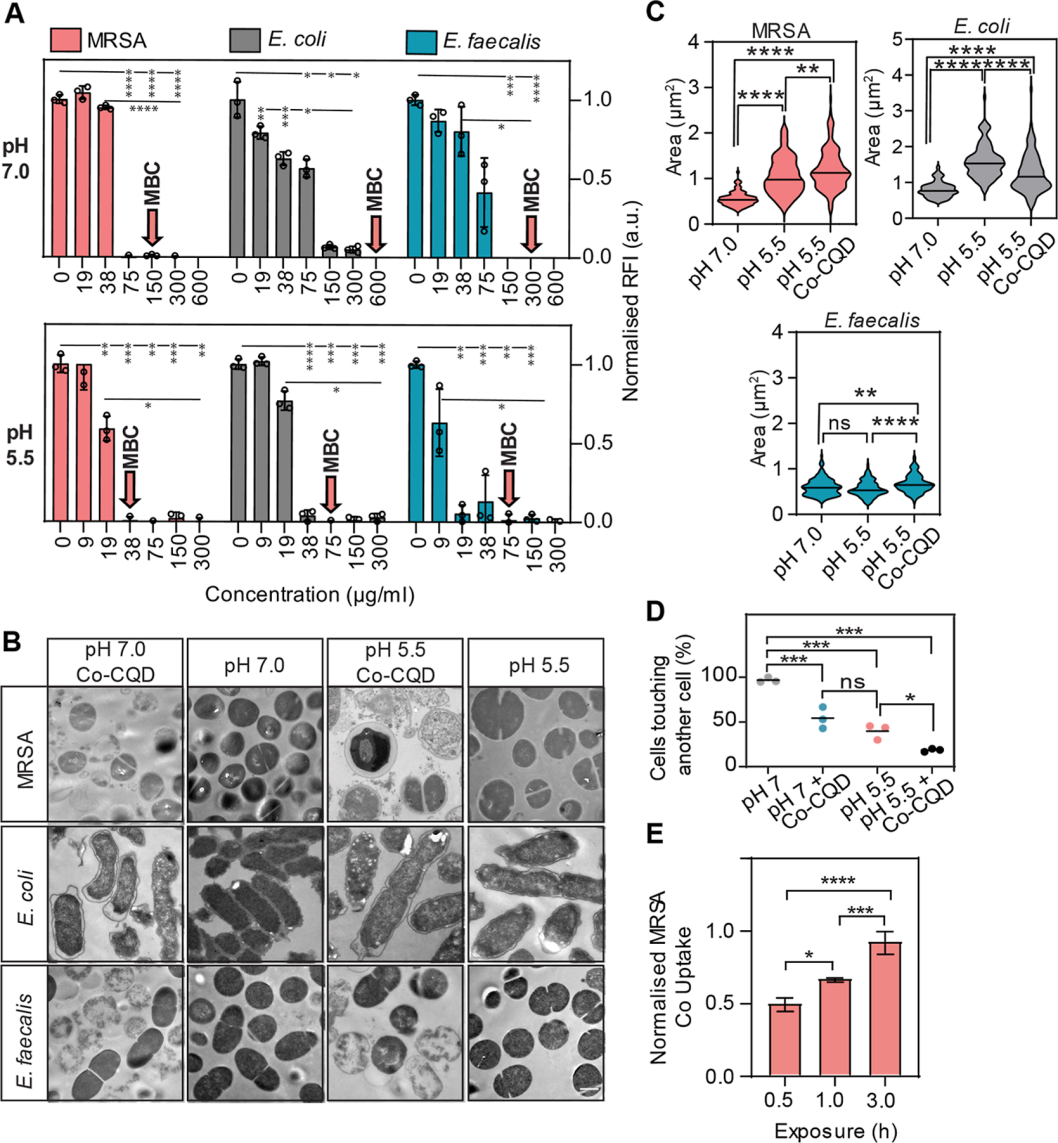
Antibacterial activity of Co-CQDs. (A) Optical density (OD_600nm_) of MRSA, *E. coli*, and *E. faecalis* in pH-adjusted liquid growth cultures after 24 h exposure to Co-CQDs. Red arrows denote the MBC Co-CQD concentration. (B) TEM images of MRSA, *E. coli*, and *E. faecalis* at pH 5.5, pH 5.5 + Co-CQDs, pH 7.0, and pH 7.0 + Co-CQDs following a 24 h exposure period. Scale bar = 500 nm. (C) MRSA, *E. coli*, and *E. faecalis* cell sizes when exposed to nutrient broth (NB) pH 7.0, NB + acetic acid (pH 5.5), and NB + acetic acid (pH 5.5) + Co-CQDs. (D) Number of *E. coli* cells that are touching another *E. coli* cell (cell–cell interaction) at pH 7 and 5.5, with and without Co-CQD treatment. (E) Normalized cobalt uptake from Co-CQD internalization in MRSA cells between 0.5 and 3 h at pH 5.5. Error bars represent the standard deviation. Statistical significance was determined using (A) a two-way analysis of variance (ANOVA) with Tukey’s multiple comparison test or a (C, D, E) one-way ANOVA with Tukey’s multiple comparison test. ns, *, **, ***, **** signifies not significant, *p* < 0.05, *p* < 0.005, *p* < 0.0005 and *p* < 0.0001, respectively.

To better understand the synergistic antimicrobial function between Co-CQDs and acetic acid, we first assessed the effect of Co-CQD on the bacterial morphology by TEM under various pHs. The addition of Co-CQDs to MRSA and *E. faecalis* cells in the acetic acid environment resulted in detachment of the cytoplasmic membrane from the outer membrane, release of cytoplasm constituents, and complete cellular disintegration (Figure 2B). *E. coli* cells exposed to Co-CQDs (in neutral and acidic conditions) had obvious detachment of the cell wall from their cytoplasm (Figure 2B). At this ultrastructural level, both MRSA and *E. coli* swelled to nearly double the size when grown at pH 5.5 compared to pH 7.0 (1.91 and 1.99 times respectively), indicating osmotic imbalance in these cells (Figure 2C). In MRSA, there was no significant difference between the ratio of cell size to peptidoglycan thickness between pH 7 and 5.5; however, as the cells were twice the size in acetic acid conditions, there was twice the amount of peptidoglycan per cell (Figure S3D). Acidic acid conditions also prevented cell–cell interactions in *E. coli*, with the percentage of cells touching a neighboring cell dropping from 96.7% at pH 7 to 39.7% at pH 5.5. The addition of Co-CQDs further reduced cell–cell interactions with 54.2% at pH 7 and 18.5% at pH 5.5 (Figure 2D). An increase in the Co-CQD uptake by MRSA cells was confirmed through cellular cobalt uptake. At both pH 5.5 and 7.0, particles were able to translocate the membrane, with particle uptake being more rapid at pH 5.5 (Figure 2E). TEM images focused on the cell membrane at 60,000× magnification show that at pH 5.5, Co-CQDs translocate through the MRSA cell membrane and into the cell (white arrows). In these conditions, *E. faecalis* cells showed Co-CQD internalization resulting in cell lysis (Figure 3A). The Gram-negative *E. coli* cell membrane seems largely impermeable to nanoparticle translocation; however, damaged membranes brought upon from the particles and the acetic acid environment presented a route for monodispersed Co-CQD entry into the cell (white arrows).

**Figure 3:**
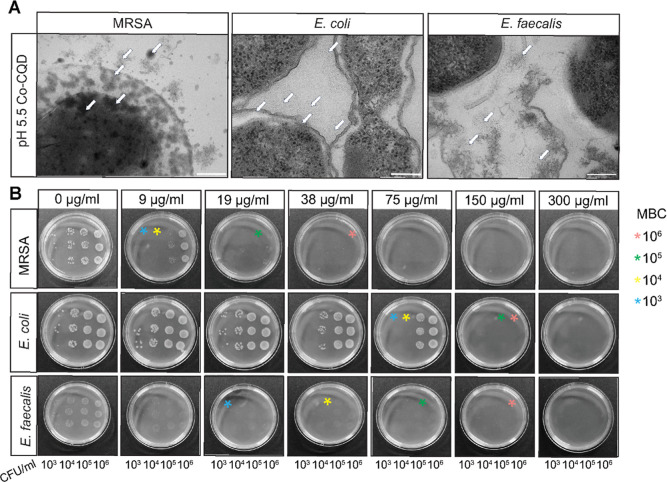
Co-CQD translocation into cells and antibacterial activity on solid surfaces. (A) TEM of Co-CQD interaction with MRSA, *E. coli*, and *E. faecalis* cell membranes at pH 5.5. Scale bar = 200 nm. (B) Growth of MRSA, *E. coli*, and *E. faecalis* on nutrient agar plates supplemented with differing Co-CQD concentrations (0–300 μg/mL) at pH 5.5 (acetic acid adjusted). Each plate has been seeded with different concentrations of bacteria (5.0 × 10^3^, 10^4^, 10^5^,10^6^ CFU/mL) from left to right in triplicate. Stars of red, green, yellow, and blue represent the MBC for 10^6^, 10^5^, 10^4^, and 10^3^ CFU/mL, respectively.

To understand how Co-CQDs influence bacterial colonization on solid surfaces, the nanoparticles were incorporated into agar plates at pH 5.5 (Figure 3B) and 7.0 (S3C).. As seen with the liquid cultures, an acetic acid environment provided enhanced antimicrobial activity (Figure 3B). The MBC for MRSA in acidic conditions was dependent on initial bacterial seeding concentration with no growth present at Co-CQD concentrations of 38, 19, and 9 μg/mL for 5.1 × 10^6^, 10^5^, and 10^4^ CFU/mL, respectively. In neutral conditions, this rose to 150 μg/mL regardless of bacterial seeding concentration (Figure S3C). The MBC for *E. coli* in acidic conditions was 150, 150, and 75 μg/mL for 6.6 × 10^6^, 10^5^, and 10^4^ CFU/mL, respectively, and for *E. faecalis* it was 150, 75, and 38 μg/mL for 5.5 × 10^6^, 10^5^, and 10^4^ CFU/mL, respectively (Figure 3B). Additionally, *E. faecalis* exhibited obvious growth suppression, as seen by faded colonies across all treatment concentrations.

These experiments thus reinforce the potent antimicrobial efficiency of Co-CQD across both Gram positive and negative bacteria, in solid and aqueous settings, particularly in a mild acetic acid environment. Acetic acid was shown to disrupt the osmotic balance of both Gram-positive (MRSA) and Gram-negative (*E. coli*) cells, causing them to swell. In this environment, the action of Co-CQDs is greatly enhanced, with Gram-positive cells actively translocating particles into the cell and Gram-negative cells allowing particle translocation through damaged membranes.

Having demonstrated potent antibacterial activity in *in vitro* assays, we next sought to assess their functionality *in vivo*. To investigate this, wounds (5 mm diameter) were created onto the back of six female black C57BL/6 mice, infected with MRSA bacteria (1.3 × 10^7^ CFU) and treated with Co-CQD nanoparticles (0.87 mg/kg) (Figure 4A). After 24 h, wounds inoculated with MRSA possessed visibly obvious infection and the presence of 6.2 × 10^8^ CFU/g (Figure S4A,B). The addition of Co-CQD nanoparticles greatly reduced the bacterial load by 4.3 log (3.0 × 10^4^ CFU/g = 99.995% reduction), indicating potent acute *in vivo* antimicrobial activity. To further assess Co-CQD pathogen control and to investigate their impact on wound healing, infected wounds were treated with Co-CQD, and wound infection and closure rates were determined over 7 days. The addition of Co-CQDs to MRSA-infected wounds (1.09 mg/kg) removed all bacterial infection (Figure 4B,C).

**Figure 4:**
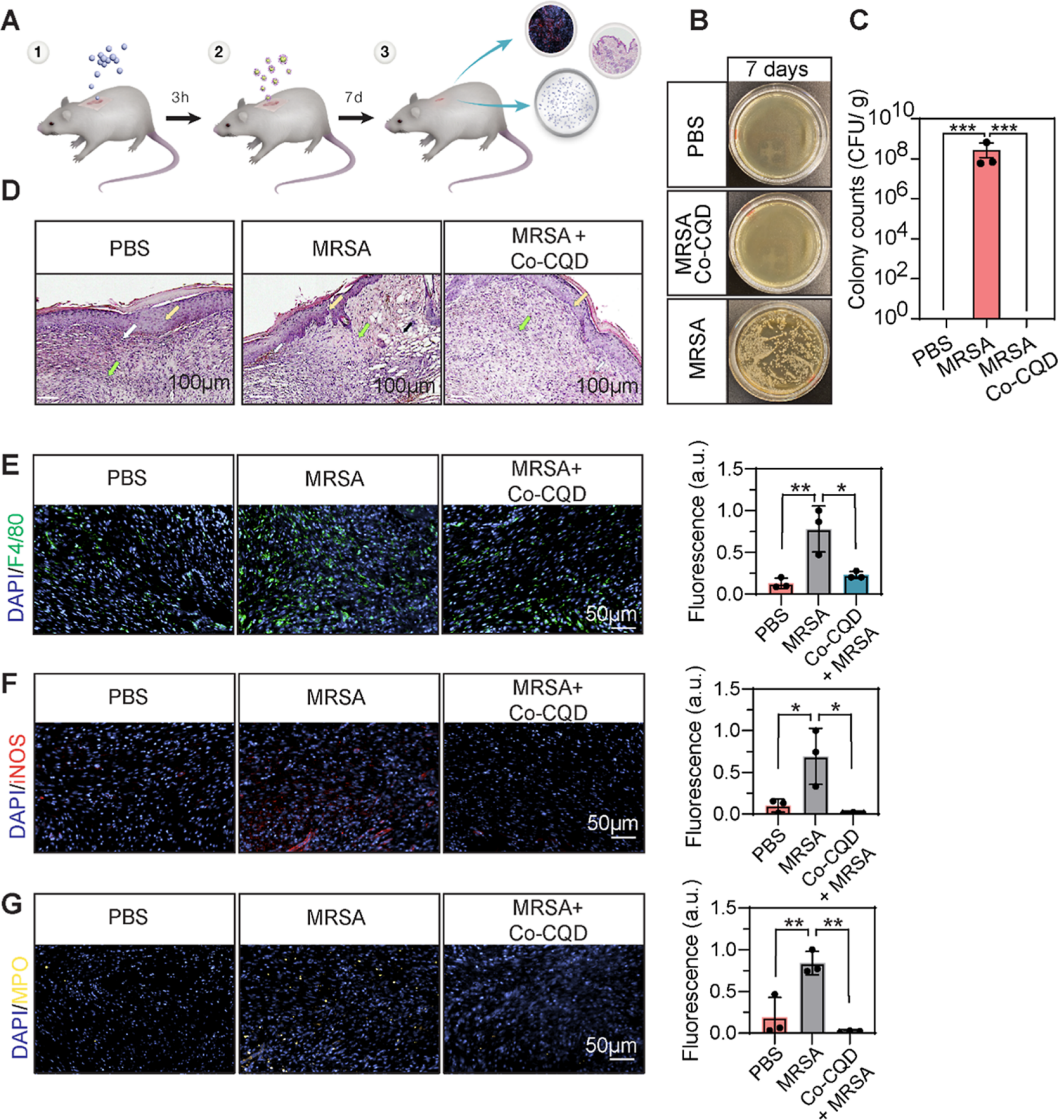
Co-CQD treatment removes infection *in vivo*, restores skin physiology, and reduces bacterial characteristic immune responses. (A) Schematic representation of *in vivo* antimicrobial treatment workflow showing bacterial infection of wounds, addition of Co-CQD nanoparticles, 7 day wound healing period followed by wound microbial load analysis, IF, and histological analysis. (B) Agar plates representing wound bacterial load after 7 d treatment exposure. (C) Quantification of bacterial presence in wound areas following treatment after 7 d. (D) Representative H&E-stained wound regions of PBS, MRSA-infected, and Co-CQD-treated MRSA-infected wounds. Green arrows denote granulation tissue, white arrows denote neovascularization, yellow arrows denote epithelium, and black arrows denote subepidermal hemorrhage tissue. (E) F4/80 macrophage presence in wound areas following treatment after 7 d. (F) iNOS inflammatory macrophage presence in wound areas following treatment after 7 d. (G) MPO neutrophil presence in wound areas following treatment after 7 d. Error bars represent the standard deviation. Statistical significance was determined using a (C, E, F, G) one-way ANOVA with Tukey’s multiple comparison test. ns, *, **, ***, **** signifies not significant, *p* < 0.05, *p* < 0.005, *p* < 0.0005 and *p* < 0.0001, respectively.

Hematoxylin and eosin (H&E) staining showed that the MRSA-infected wounds possessed subepidermal hemorrhage tissue areas (black arrows), nonuniform granulation (green arrows), and an incomplete epidermis layer (yellow arrows) despite also possessing clear neovascularization (white arrows) (Figure 4D). This tissue damage suggests that wound inflammation and immune presence are active, which may be associated with increased MRSA presence. Treatment with Co-CQD nanoparticles resulted in normalization of the wound areas including abundant granulation tissue (green arrows), structurally complete epidermis layers (yellow arrows), and clear neovascularization (white arrows) (Figure 4D).

Immunofluorescence staining demonstrated that Co-CQD treatment of MRSA-infected wounds contained significantly reduced macrophage (F4/80, Figure 4E), inflammatory macrophage iNOS, (Figure 4F) and neutrophil (MPO, Figure 4G) positive cells, indicating decreased inflammation as a result of reduced bacterial infection. As the presence of pathogenic bacteria increases iNOS activity,^32–34^ its decreased presence in Co-CQD-treated groups supports their antimicrobial activity. There was no significant difference in F4/80, iNOS, and MPO-positive cells between the Co-CQD treatment and the PBS control.

Combined, these findings demonstrate that the particles removed drug-resistant infection from mice wounds, resulting in reduced bacterial characteristic immune presence leading to a skin pathology comparable to the uninfected healing wounds.

### Acetic Acid Primes MRSA for Co-CQD-Dependent Oxidative Damage

Having demonstrated the entry into the bacteria, we next set out to determine the mechanisms underpinning the bactericidal effects of Co-CQD treatment. To this end, we next performed RNA sequencing on bacteria exposed to pH 7.0, 5.5, and 5.5 with Co-CQDs. The volcano plot in Figure 5A highlights that MRSA exposed to acidic conditions responded by upregulation of 84 and suppressed expression of 87 genes. When Co-CQDs were added to these acidic conditions, there were further 53 upregulated and 67 downregulated genes. Pathways analysis of MRSA in an acetic acid environment highlighted repressed genes belonging to oxidative phosphorylation, indicating that ATP generation was suppressed, resulting in reduced cellular respiration (Figure 5B). This was supported by a decreased metabolic activity measured by the cells’ inability to convert non fluorescent resazurin to fluorescent resorufin by dehydrogenase enzymes found in metabolically active cells (Figure 5C). Genes relating to hydrogen ion transport were also repressed as an adaptive measure for tolerating the acidic environment.

**Figure 5:**
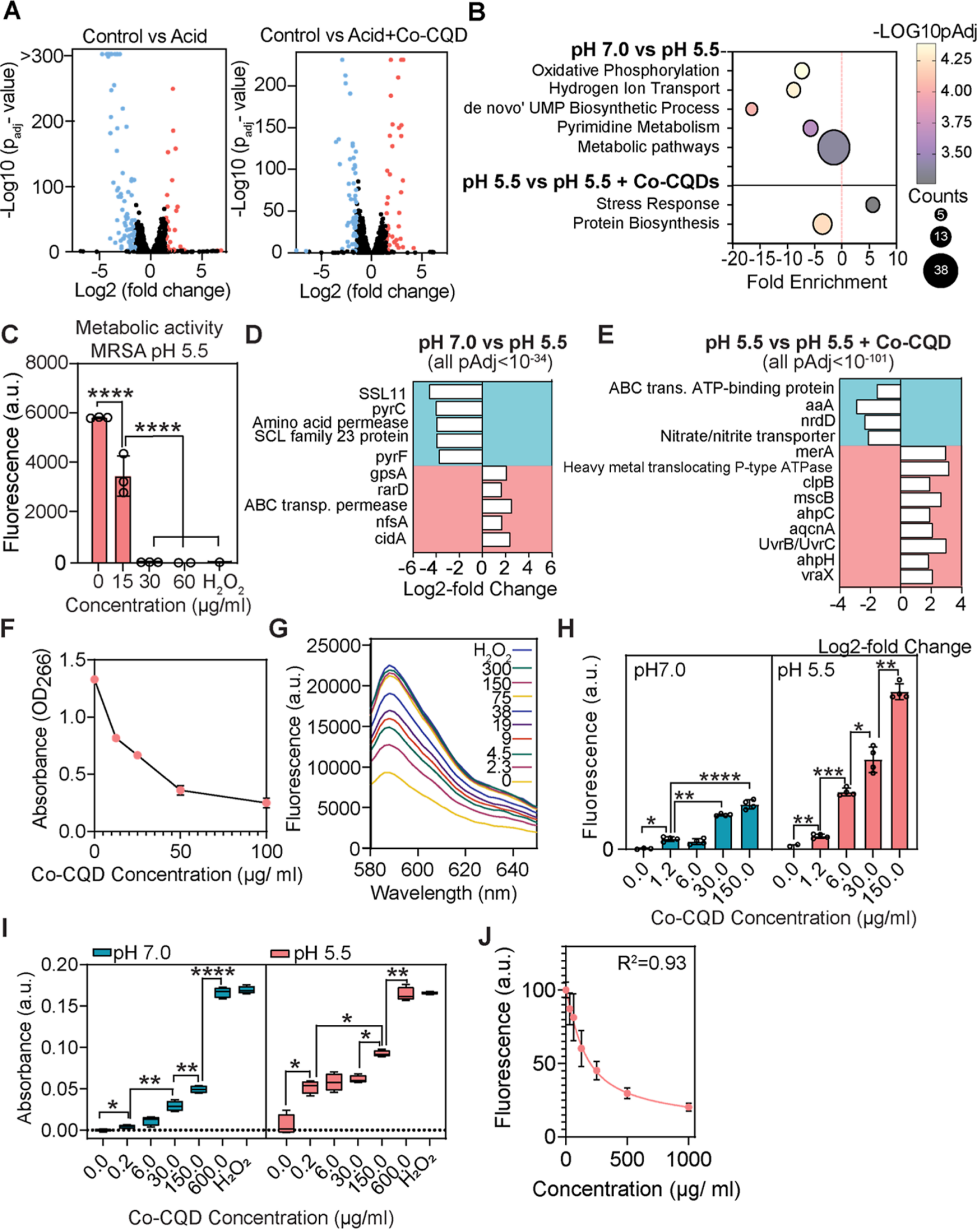
Mechanism of Co-CQDs’ antibacterial activity. (A) Volcano plots of differentially expressed genes between MRSA subjected to pH 7.0 vs pH 5.5 (acetic acid adjusted) and differentially expressed genes between MRSA subjected to pH 5.5 vs pH 5.5 + Co-CQDs. (B) Pathways analysis plot of MRSA subjected to pH 5.5 vs pH 5.5 + Co-CQDs. (C) Metabolic activity (resazurin conversion to resorufin) of MRSA when subjected to Co-CQDs at different concentrations at pH 5.5 (acetic acid adjusted). (D) Expression of significant genes when MRSA is transferred from pH 7.0 to pH 5.5 (acetic acid adjusted). (E) Expression of significant genes when MRSA is transferred from pH 5.5 (acetic acid adjusted) to pH 5.5 containing Co-CQDs. (F) ROS generation determined through the oxidation of ascorbic acid (AA) at pH 7.0. (G) Hydrogen peroxide generation of Co-CQDs measured via the Amplex Red assay. (H) Co-CQDs’ superoxide generation capacity and its interaction with nucleic acids in a pH 7.0 and 5.5 environment. (I) Alkaline phosphatase (AlkP) activity indicating MRSA membrane damage at pH 7.0 and 5.5 from Co-CQD exposure. (J) Membrane hyperpolarization of MRSA at pH 5.5 resulting from differing Co-CQD exposure. Error bars represent the standard deviation. Statistical significance was determined using (H, I) a two-way ANOVA with Tukey’s multiple comparison test or a (C) one-way ANOVA with Tukey’s multiple comparison test. ns, *, **, ***, **** signifies not significant, *p* < 0.05, *p* < 0.005, *p* < 0.0005, and *p* < 0.0001, respectively.

The addition of Co-CQD to MRSA in an acetic acid environment increased the regulation of bacterial stress responses and decreased the protein biosynthesis pathways (Figure 5B). Of the genes identified in the stress response pathway, DnaK, DnaJ, and GrpE chaperones, Clp ATPases (clpP and clpB), and CtsR and HrcA regulons have been implicated in cellular protection from oxidative stress.^35–37^

At the single gene level, when MRSA was exposed to an acetic acid environment, several genes responsible for drug exporting, antibiotic resistance, and chemical detoxification were upregulated (EamA family transporter (RarD), ABC transporter permease, NAD­(P)-dependent oxidoreductase, oxygen-insensitive NADPH nitroreductase, etc.), indicating that the cells were trying to establish a new favorable equilibrium in the adverse acidic environment (Figure 5D).

When Co-CQD nanoparticles were added to the acetic acid growth media, enhanced bacterial stress responses were observed. Several genes responsible for the protection against oxidative stress were upregulated (ATP-dependent chaperone (ClpB), protein arginine kinase (mcsB), alkyl hydroperoxide reductase subunit C (ahpC), hypothiocyanous acid reductase (MerA), aconitate hydratase (acnA)), indicating that the particles were generating ROS (Figure 5E). In addition, alkyl hydroperoxide reductase subunit F (ahpF), was upregulated, which is responsible for protecting DNA from peroxides, and C1q-binding complement inhibitor VraX emphasizes that cell wall stress was apparent due to Co-CQD particles. Several heavy metal translocating genes were upregulated (heavy metal translocating P-type ATPase, CDF family zinc efflux transporter CzrB, Zn­(II)-responsive metalloregulatory transcriptional repressor CzrA, and cadmium-translocating P-type ATPase CadA), indicating a metal efflux strategy was being employed to detoxify their environment.

Taken together, these results indicate that an acetic acid environment causes cell stress and, as a result, adapts by switching on numerous detoxification strategies to prevent oxidative membrane and DNA damage. The addition of Co-CQDs further stressed the cell, with genes responding to oxidative stress being induced.

### Co-CQD Nanoparticles Cause Multiple Stresses on MRSA Cells in an Acetic Acid Environment

Buildup of cellular reactive oxygen species is closely linked to lipid peroxidation and cell wall damage, consistent with our ultrastructural TEM and RNA sequencing conclusions. Given genes relating to combatting oxidative stress, in particular, peroxides were upregulated in Co-CQD NP-treated samples in acetic acid conditions (i.e., protein arginine kinase (mscB), alkyl hydroperoxide reductase (ahpC) (Figure 5E)), we next set out to phenotypically verify the transcriptional data. The oxidation of the antioxidant AA in the presence of Co-CQD was initially measured. At concentrations between 12.5 and 100 μg/mL the addition of Co-CQD nanoparticle resulted in the dose-dependent oxidation of AA by its reaction with ROS or autoxidation by cobalt (Figure 5F). Consistently, when treated, bacteria were subjected to an “Amplex Red” assay, which measures hydrogen peroxide generation through the oxidation of 10-acetyl-3,7-dihydroxypenoxazine by peroxides in the presence of a horseradish peroxidase catalyst. The formation of an increasingly red, fluorescent oxidation product was apparent with a stepwise increase with increasing nanoparticle concentration between 2.3 and 300 μg/mL, verifying the production of peroxides (Figure 5G). In addition to peroxide generation, the particles were shown to produce superoxide anions (O_2_^•^) through the fluorescent products of DHE oxidation by a superoxide (Figure 5H). Acetic acid conditions increased the concentration of superoxide formed, and superoxide dismutase, responsible for converting superoxide into H_2_O_2_ and O_2_, was upregulated in acidic conditions (2.2-fold change, 43.0 – Log­(*P*)­Adj). These experiments thus highlight that the Co-CQD particles potently induce multiple reactive oxygen species.

AlkP is produced and localized in the cell walls and cell membranes of bacteria. The detection of extracellular AlkP is therefore a measure of cell wall damage after the ROS damage. At both pH 7 and pH 5.5, there was an increase in extracellular AlkP with increasing nanoparticle treatment with 0, 6, 30, 150, and 600 μg/mL showing AlkP-dependent absorbance values of 0.00, 0.052, 0.055, 0.061, 0.092, and 0.164, respectively (Figure 5I). Bacterial cell wall damage was increased under acetic acid conditions (pH 5.5) with an additive effect from nanoparticle addition. At 600 μg/mL treatment concentration, the extracellular AlkP in the system was comparable to that of cells exposed to 15 mM H_2_O_2_ with both showing absorbances of 0.164 and 0.166, respectively. At pH 7, AlkP levels also increased with increasing nanoparticle concentration with 0, 6, 30, 150, and 600 μg/mL showing absorbance values of 0.000, 0.004, 0.011, 0.029, 0.049, and 0.166, respectively (Figure 5I).

Finally, bacterial membrane potential properties were determined using the cationic DiSC_3_(5) dye, which quenches when it translocates and binds to the lipid bilayer of hyperpolarized membranes. Hyperpolarized membranes are increasingly negative in charge and hamper ion flux and intracellular signaling processes. This negative cellular surface charge may be due to carboxylic acid functional groups on Co-CQD interacting with the cell. At pH 7 increased Co-CQD treatment resulted in cells possessing increasingly negative membrane potential (hyperpolarization) resulting in stepwise fluorescence quenching of the DiSC_3_(5) dye compared to the untreated controls (Figure S5A). At pH 5.5, this followed a logarithmic concentration-dependent trend, with a trendline showing *R* ^2^ values of 0.93 (Figure 5J).

Overall, Co-CQD nanoparticles cross the MRSA cell membrane due to their relatively neutral charge, and osmotic imbalance created from the presence of acetic acid, which draws in water (and Co-CQDs), resulting in cell swelling. In the acidic environment, the particles generate ROS, particularly peroxide in nature but also superoxide, and attack the cell from inside and out.

### Co-CQDs Are Biocompatible *In Vitro* and *In Vivo*

Having demonstrated the bactericidal effects of Co-CQDs on bacterial strains, we then asked how Co-CQD affects mammalian cells and physiology. When Co-CQDs were added to wounds (1.09 mg/kg), there was no significant difference in mouse body weights over 7 days, supporting the particles’ biocompatibility (Figure 6A). Further, representative images of wound closure over time shown in Figure 6B demonstrated that addition of the Co-CQD had no visible toxicity on the wound healing process (Figure 6B,C). Combined, this suggests that the Co-CQD were well tolerated by the mice. We next sought to assess their influence at a cellular level. During wound healing, fibroblasts generate collagen for promoting connective tissue formation.^38^ The biocompatibility of the Co-CQD particles was assessed in human dFIB at pH 7.0 and 5.5 at concentrations of 10, 50, 100, and 500 μg/mL. Human fibroblasts were resilient to Co-CQD treatments in both neutral and acidic environments. At pH 7.0, dermal fibroblast cells had >96% viability between 0 and 500 μg/mL; however, at 500 μg/mL, the cells were smaller in size. At pH 5.5, Co-CQD treatments up to 100 μg/mL showed >95% viability, while at 500 μg/mL, the viability dropped to 76%. It is worth noting that at pH 5.5, cells did not proliferate but they did not die either. Once the acidic media (with and without Co-CQDs) were replaced with fresh unamended growth media, the cells were able to recover and proliferate (Figure 6G). This indicates that mammalian cells entered a quiescent state awaiting favorable conditions for proliferation, with recovery growth rates indicating that their proliferative potential was not compromised. It is worth noting that lower-molecular-weight organic acids such as acetic acid (1%) has been shown to have a short lifetime in wounds (1 h), before the pH returns to pretreatment levels.^20^ Therefore, the extreme 24 h exposure time in our experiments is unlikely to be experienced under *in vivo* conditions. An MBC of 38 μg/mL Co-CQDs was determined for MRSA in an aqueous acetic acid environment, but dFIB were much more resilient, showing 76% viability at >13-fold increased nanoparticle concentrations (500 μg/mL). Considering the high tolerance of dFIB to Co-CQD treatment *in vitro*, we sought to assess if this also applied to *in vivo* models. Infected mice wounds treated with Co-CQDs were allowed to heal for 7 days, and activated fibroblast-derived fibrosis was measured through collagen 1 deposition in treated wound areas. In MRSA-infected wounds, there was a significantly reduced collagen 1 deposition compared to the PBS control and Co-CQD-treated MRSA-infected wounds (Figure 6H,I). This supports findings that MRSA wound infection impairs fibroblast function.^39^ The Co-CQD-treated wounds showed fibrosis content similar to that of the PBS control (Figure 6H,I), indicating that the treatment did not impair normal fibroblast function and collagen production.

**Figure 6:**
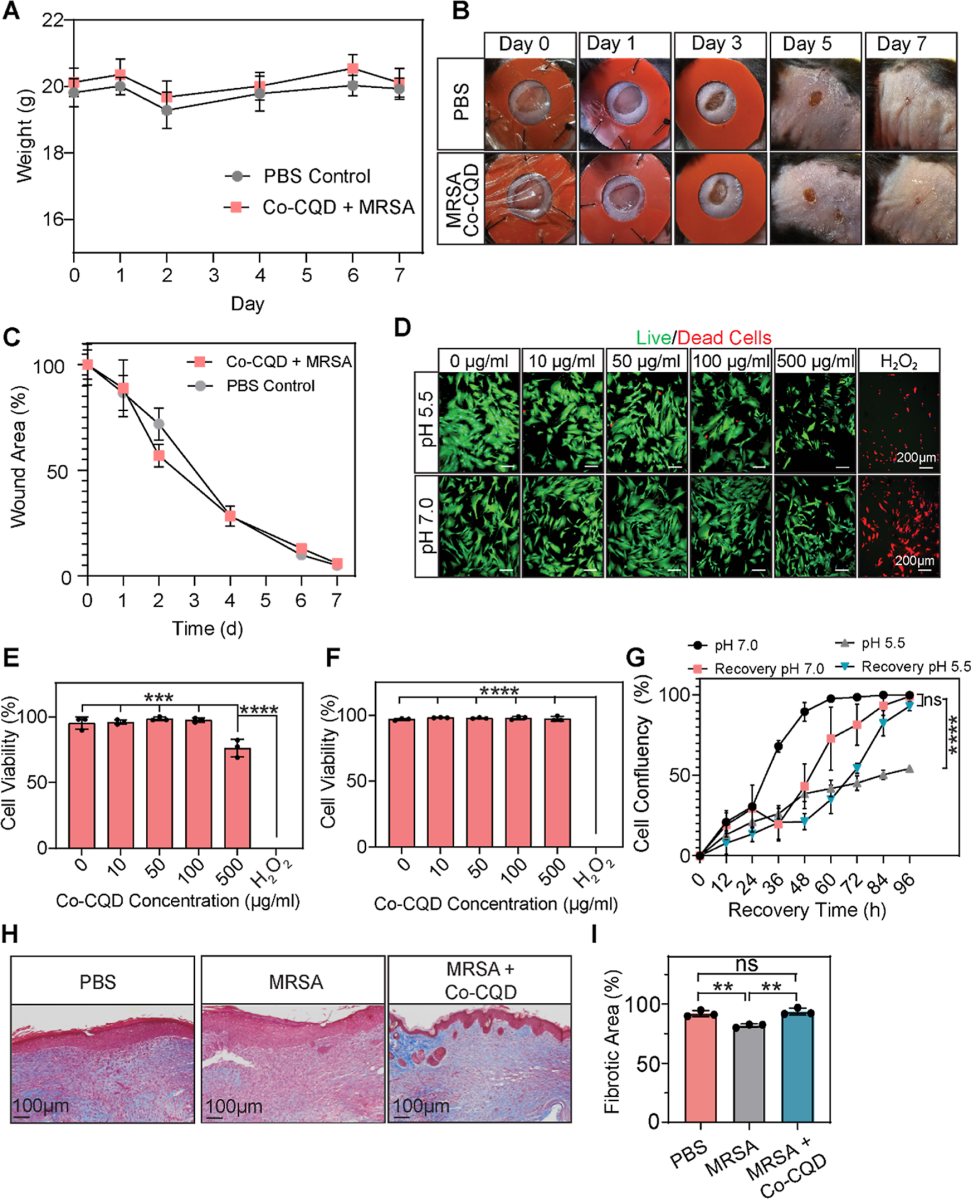
Co-CQD biocompatibility. (A) Mouse weights over 7 days after inflicting full thickness wounds (5 mm diameter) in black 6 mice with and without Co-CQD treatment. (B) Representative images of full thickness wounds in black 6 mice, treated with PBS or infected with MRSA and then treated with Co-CQDs for 7 d. (C) Quantification of wound closure following PBS- and Co-CQD-treated MRSA wounds over a 7 d time period. (D) Representative fluorescent confocal microscope images of human dFIB in the presence of differing Co-CQDs concentrations at pH 7.0 and 5.5. The live/dead probes fluoresce green in viable cells and red in dead cells. (E) Graphical quantification of aforementioned cell viability at pH 5.5 and (F) at pH 7.0 when subjected to Co-CQDs at different concentrations over 24 h, respectively. (G) Cell proliferation (confluency over time) of human dFIB subjected to pH 7.0, pH 7 + Co-CQDs (500 μg/mL), pH 5.5, and pH 5.5 + Co-CQDs (500 μg/mL) for 24 h followed by their recovery when growth media was replaced with unamended media following 24 h of acetic acidic exposure (pH 5.5). (H) Trichrome stain (blue) of mouse wound tissue following 7 days treatment showing the presence of collagenous connective tissue fibres. (I) Area of collagenous connective tissue fibres present in mouse wound tissue following 7 days treatment. Error bars represent the standard deviation. Statistical significance was determined using (I) a one-way ANOVA with Tukey’s multiple comparison test or an (E, F, G) unpaired *t*-test with Welch correction. ns, *, **, ***, **** signifies not significant, *p* < 0.05, *p* < 0.005, *p* < 0.0005 and *p* < 0.0001, respectively.

Combined, the addition of Co-CQD to wounds *in vivo* and dermal fibroblast *in vitro* demonstrated negligible toxicity. As such, Co-CQD is a viable and effective MSRA infection control strategy.

## Discussion

The Co-CQD produced in this study are classified as low-dimensional antimicrobial nanomaterials (LDMs). LDMs include zero-dimensional (0D) materials (all dimensions <10 nm, e.g., carbon quantum dots or nanodiamonds), one-dimensional (1D) materials (two dimensions <10 nm, often forming nanotubes or nanowires), and two-dimensional (2D) materials (one dimension <10 nm, such as graphene sheets).^40^ All forms have demonstrated antimicrobial properties; for example, Wang et al. (2023) created 0D Cu,N-doped graphene quantum dots with photoactive ruthenium nitrosyl nitric oxide donors that effectively destroyed MRSA, inhibiting bacterial growth at 500 μg/mL when irradiated with an 808 nm laser.^41^ In comparison, the 0D Co-CQDs from this current study fully eliminated MRSA, *E. coli*, and *E. faecalis* at 38, 75, and 75 μg/mL, respectively, at pH 5.5 without requiring the use of external aids (i.e., infrared lasers).

Both 1D and 2D nanomaterials typically exert antimicrobial effects through chemical and/or physically induced damage to microbial membranes.^40,42,43^ While physical membrane piercing and slicing is an effective strategy, these surfaces can be fouled easily in a wound environment, reducing their ongoing antimicrobial efficacy. Furthermore, these materials may also harm mammalian cells due to their nonspecific mode of action.^44^ He et al. developed 1D nitrogen-doped carbon nanotubes with encapsulated cobalt nanoparticles that effectively deactivated *S. aureus* via oxidase-like activity, producing bactericidal ROS and nearly eliminating bacterial growth at 60 μg/mL. The particles were also biocompatible with L929 immortalized fibroblasts cells up to 100 μg/mL.^45^ The study showed that cobalt-doped carbon nanomaterials can produce strong bactericidal activity. The Co-CQDs generated in the present study produced peroxidase like and superoxide ROS activity to completely eliminate *S. aureus* at a 38 μg/mL concentration in a weak acetic acid environment. Furthermore, they showed 76% viability of primary dermal fibroblast cells at 500 μg/mL treatment concentration, and these cells could resume healthy proliferation rates when reintroduced to fresh growth medium following 24 h of exposure.

Perreault et al. (2015) studied 2D graphene oxide nanosheet size and antimicrobial effects on *E. coli*. Small sheets destroyed bacteria via oxidation on surfaces at 200 μg/mL, while large sheets in liquid only wrapped bacteria without killing them.^46^ Within the current study, the Co-CQDs were zero-dimensional, monodisperse, and almost neutral in charge, which facilitated their passive diffusion within bacterial cells with the aid of acetic acid, allowing microbial attack from both outside and within the cell.

Recent advances in non-LDM nanoparticle-based therapies have enabled the antibiotic-free removal of MRSA wound infections. Li et al. (2024) created a combination therapy using Fe- and N-doped carbon nanozymes that effectively killed *S. aureus* (1 × 10^6^ CFU/mL) with H_2_O_2_ and near-infrared light. At 125 μg/mL, this treatment nearly eradicated *S. aureus* under 808 nm laser irradiation.^47^ The use of hydrogen peroxide as an antiseptic has recently been considered unfavorable, as it irritates the skin and damages both bacterial and mammalian cells alike, slowing wound healing. In contrast, acetic acid is a simple, cheap, and safe topical antiseptic shown to promote reepithelialization and disperse bacterial biofilms. It possesses strong antimicrobial activity against *P. aeruginosa*; however, its effectiveness against other microbial strains varies, and as a result, it is not commonly used clinically.^48^ Findings from this current study show that its use in combination with biocompatible Co-CQDs results in synergistic bactericidal activities against Gram-positive and Gram-negative species, not typically sensitive to acetic acid alone.

Silver nanoparticles are the most common antimicrobial nanoparticles commercially used due to their strong activity and stability for integration into fibers and matrices. For stable integration and to prevent particle agglomeration, which reduces their efficiency, surface modification is needed. Reported antimicrobial concentrations (MBCs) against MRSA vary widely, from under 12.5 μg/mL^49^ to over 2.7 mg/mL^50^ at cell densities above 1.0 × 10^6^ CFU/mL. Unlike metals, carbon has a unique capacity to bind to itself and other elements, increasing its potential to functionalize surfaces. The Co-CQDs produced in this study were water-soluble, monodisperse without postsynthesis modification, and only contained a 2.31 at. % metal dopant concentration but showed antimicrobial activities approaching those of silver nanoparticles with the highest antimicrobial capacities.

The Co-CQDs strategy developed here is intrinsically versatile and could be extended to clinical applications beyond wound healing, provided that systemic toxicity and bioaccumulation are shown to be negligible. For example, they could be integrated into surgical sutures, orthopedic implants, or hernia meshes to prevent hospital-acquired infections during and postsurgery. Furthermore, the use of nanoparticle-based antimicrobial agents either alone or in combination with traditional chemical antibiotics will help to curb the threat of antimicrobial resistance. There is also potential for their use in systemic therapeutic applications. Their small size coupled with abundant polar functional groups give them water solubility and may improve drug solubility when bound via solid dispersion,^51^ increasing their therapeutic potential. In addition, with particle sizes below 5 nm and low surface charge, the likelihood of renal filtration is high, preventing bioaccumulation and the need for the body to break them down.^52^

## Study Limitations

It is important to note that wound healing in mice differs fundamentally from that in humans. Mice wounds contract during healing while human wounds close predominately through re-epithelization.^53^ While this represents a limitation of the present study, efforts were made to minimize contraction during the initial stages of wound healing, by splinting the wound, which promotes re-epithelization. In future works, pig models could be considered as they more closely replicate human wound-healing dynamics.

In this study, we assessed the particles’ capacity to remove infection at the wound site and terminated the study once wounds had closed after 7 days. However, evaluation of mouse health over longer periods of time would further validate the treatment effectiveness, including its potential to prevent sepsis or bacterial bloodstream infections and help identify any delayed adverse effects. A longer exposure study exploring particle distribution from the wound site to the bloodstream, for instance, as well as their metabolism and excretion, would be valuable. This could be followed by a comprehensive study investigating the effects of particles on mouse organs, including systemic toxicity and assessment of changes in blood biomarkers in both the presence and absence of bacterial infection.

## Conclusion

For nonhealing infected wounds, topical antibiotics are rarely used as they can cause inflammation, adverse skin reactions, and increase the risk of antimicrobial resistance.^54^ Furthermore, their additive contribution to pathogen control in surgical site infections and nonhealing wounds has been questioned.^55^ It is suggested that treatment of these wounds requires systemic antibiotics used together with topical antiseptics.^54^ In the present study biocompatible carbon quantum dot nanoparticles doped with cobalt were designed as potent topical antimicrobial agents against several bacterial pathogens including multidrug-resistant Gram-positive MRSA, Gram-negative *E. coli*, and vancomycin-sensitive Gram-positive *E. faecalis*. The particles exhibited strong antimicrobial activity against planktonic and surface-colonized bacteria. In addition, the study demonstrates the impact of an acetic acid antiseptic on bacterial cell fitness and shows that when Co-CQDs and acetic acid are used together, a synergistic and greatly enhanced antimicrobial activity is achieved.

Healthy healing wounds exhibit a fluctuating pH, but pathogen infections typically create a persistent alkaline environment, slowing the healing process. Wound acidification has therefore been considered a potential treatment for alleviating wound infection. In addition, an acidic environment has been shown to result in higher wound oxygen content and increased angiogenesis and can transform bacterial end-products to less toxic forms (i.e., ammonia), promoting wound healing. In an acetic acid environment, MRSA and *E. coli* cells swelled and their osmotic balance was disrupted, increasing the speed at which Co-CQDs could enter the cell. These particles generated peroxidase and superoxide ROS, causing MRSA and *E. faecalis* to burst and *E. coli* to have their cell wall detach from the cytoplasm. Pathways analysis shows that Co-CQDs suppressed bacterial protein translation and increased stress responses, mainly through oxidative damage. These findings were reinforced through cell swelling observations, extracellular AlkP presence, capacity to generate peroxidase/peroxide, and AA oxidation by ROS. The particles were shown to be biocompatible with human dFIB at concentrations >13-fold the MBC of MRSA in an acetic acid environment. When applied to mice, the particles removed MRSA infection and allowed wounds to heal at the same rate as uninfected wounds.

Here we demonstrate a biocompatible Co-CQDs antimicrobial platform that destroys Gram-positive and Gram-negative bacteria through the synergistic action of acetic acid and Co-CQD nanoparticles. Acetic acid passively enters the cell, facilitating the entry of ultrasmall, monodispersed Co-CQDs and causes microbial cell osmotic imbalances. In this environment, the particles generate damaging peroxidase-like activity and superoxide radicals that act both externally and from within the cell.

## Materials and Methods

### Synthesis of HACC CQDs

Citric acid (5.4 g) and hexamine cobalt chloride (1.1 g) were dissolved in 30 mL of Milli-Q water, and the solution was transferred to a poly­(tetrafluoroethylene)-lined autoclave (50 mL). Vessels were heated at 205 °C for 2 h and cooled to room temperature. The resulting red solution was purified via dialysis (100–500 MWCO) against ultrapure water (18.2 MΩ·cm resistivity) for 50 h with regular water changes. The purified particle solution was subjected to lyophilization to obtain a fluffy, light purple powdered product.

### Characterization of CQDs

TEM images were obtained using a Hitachi HT7800 between 80 and 100 kV. Ultraviolet–vis absorption and fluorescence spectra were recorded using a Tecan SPARK using 96-welled plates. For absorbance readings below 300 nm, a quartz cuvette was used in conjunction with an Agilent 8453 UV–visible spectroscopy system. FT-IR spectroscopy was measured using a Nicolet Protege 460 spectrograph between 400 and 4000 cm^–1^. Zeta potentials were measured using a Malvern 2000 Zetasizer within a DTS 1060C cuvette (Malvern) in Milli-Q water. To determine the nanoparticles’ cobalt concentration (wt %), particles were digested in 2:1 nitric acid (70%)/sulfuric acid (99%) solution at 100 °C for 10 h, followed by the slow addition of H_2_O_2_ (0.5 mL of 35% concentration) followed by 4 h of further digestion. Particles’ cobalt concentration was analyzed with HR-ICP-MS ElementXR, Thermo Fisher Scientific. X-ray photoelectron spectroscopy was run on a Thermo Scientific K-Alpha XPS instrument with a monochromated Al 1487 eV Kα source. Peak fitting and background subtraction were interpreted using a Shirley background model using CasaXPS 2.3.23 software. Raman spectra were acquired on a Horiba Jobin Yvon LabRAM-HR800 system. The crystallinity of Co-CQDs was determined using a Bruker D8 Advance Eco X-ray diffractometer with a cobalt X-ray source. All X-ray data were obtained in the θ – 2*u* locked-couple mode over a 2θ interval of 10–90°.

### Microbiological Components

#### Strains and Media

Methicillin- and oxacillin-resistant *S. aureus* (MRSA, ATCC 43300), *E. coli* (ATCC 25922), and vancomycin-sensitive *E. faecalis* (ATCC 29212) were routinely cultured in NB/agar (NA) or brain/heart infusion media.

#### Antimicrobial Assays (Liquid Culture) (pH Dependence) Metabolic Activity (Resazurin) and MIC

Overnight cultures of bacteria were reinoculated into fresh NB (1%) and grown to an exponential phase. NB (pH 5.5, 7.0, and 8.5) with differing Co-CQD treatment concentrations (300 to 9 μg/mL) were inoculated with exponentially growing bacteria (*E. coli* (2.65 × 10^6^ CFU/mL), *S. aureus* 1.23 × 10^6^ CFU/mL, *E. faecalis* 1.35 × 10^6^ CFU/mL, and grown for 24 h at 37 °C. Bacterial growth was determined by measuring absorbance at OD_600_). To determine the MBC, 10 μL of each growth culture was removed following the 24 h period, spread onto NA, and incubated at 37 °C. Following 48 h of growth, plates were assessed for colony growth.

To identify the metabolic activity of the growth culture, 30 μL of resazurin solution (0.015% w/v) was added to 100 μL of each 24 h exposed treatment. Following a 3 h incubation, the fluorescence of the solution was measured at ex 560 and em 590 nm. For samples with a pH of 5.5, the pH was adjusted by adding additional growth media (pH 7) prior to adding resazurin. This is because the acidic pH discolors the resazurin, leading to inaccurate results.

%⁡Reduction of resazurin
reagent=(experimental RFU value−negative control RFU value)/((100%reduced
positive control RFU values−negative control RFU values)−negative control RFU value)×100

### Antimicrobial Assays (Solid Growth)

#### Growth on Plates with Different Initial Bacterial Seeding Concentrations

Overnight cultures of bacteria were reinoculated into fresh NB (1%) and grown to the exponential growth phase. A 10 μL aliquot of bacterial culture (*E. coli* (6.65 × 10^6^ CFU/mL), *S. aureus* 5.11 × 10^6^ CFU/mL, *E. faecalis* 5.46 × 10^6^ CFU/mL) was added to nutrient agar plates (pH 7.0 and 5.5) containing Co-CQD nanoparticles (300 to 9 μg/mL) in three independent replicates. Serial dilutions of the bacteria were added to the agar plate in the same manner to generate a concentration-dependent viability assay (10^6^, 10^5^, 10^4^, 10^3^ concentrations).

### TEM for HACC CDs on Bacterial Cells

Samples were fixed in 2.5% glutaraldehyde (diluted in a 0.1 M sodium cacodylate buffer) and 2% paraformaldehyde for 24 h at 4 °C. Post fixation was performed for 1 h (on ice) in 1% osmium tetroxide (EMS no. 19134) diluted in 0.1 M sodium cacodylate buffer, followed by 2 washing steps. The samples were then dehydrated using a graded ethanol series (30%, 50%, 70%, 96%, and 100%) before being transferred to a 1:1 solution of 100% ethanol/propylene oxide (15 min). Samples were then transferred to 100% propylene oxide (15 min) before gradually introducing agar 100 resin (Agar Scientific R1031) drop by drop over the next hours. Samples were then transferred to a small drop of 100% resin, and excess propylene oxide was allowed to evaporate (1 h). Samples were then transferred to 100% resin, placed in molds, and left at room temperature overnight. The molds were placed at 60 °C for 48 h to polymerize. Ultrasections of approximately 60 nm were placed on 100 mesh formvar-coated (EMD no. 15820) copper grids (EMS no. G100H-Cu) and stained with 2% uranyl acetate (EMS no. 22400) and lead citrate (VWR no. 1.07398). Grids were imaged using a Hitachi HT7800 transmission electron microscope at 100 kV.

### Bacterial Cell Size

The width and height of all intact bacteria imaged under TEM (at least 3 images from independent samples per strain, treatment, and pH) were manually measured using measurement tools from the Hitachi EMP-EX software. Peptidoglycan thickness from Gram-positive species was also measured.

### Cell–Cell Interactions (*E. coli*)

From TEM images, each *E. coli* cell was recorded as either touching another cell or not. The number of cells that were touching at least one other cell was represented as a percentage of total cells per TEM image (at least 3 images from independent samples for *E. coli* per treatment and pH).

### RNA-Seq Data Analysis

Methicillin- and oxacillin-resistant *S. aureus* were grown in unamended NB to an absorbance of OD_600_ = 2.5. This solution was transferred into sterile 1 mL tubes and centrifuged, and the supernatant was removed. The bacteria were centrifuged, and the supernatant was removed. The bacterial pellet was resuspended with 1 mL of the following solutions and incubated at 37 °C for 3 h:

1. NB unamended
2. NB pH 5.5 (acetic acid adjusted)
3. NB pH 5.5 (acetic acid adjusted) + Co-CQD (600 μg/mL)

RNA was extracted using TRIzol Max Reagent and a Phasemaker Tubes Complete System (A33251) as per the manufacturer’s instructions (Pub. No. MAN0016163, Rev. A.0). Crude RNA was purified twice using RNA Clean & Concentrator-25 kit (Zymol). Ribosomal RNA removal, cDNA library construction, and paired-end sequencing were performed using the NextSeq 500 platform (150 cycles) (2 × 75 bp paired end reads), ca. 100 M read pairs by NorSeq Sequencing core (Ullevål) (Oslo University Hospital, Norway).

Sequenced reads were quality checked with FastQC and aligned to the Refseq reference genome (GCF_003052445.1) using Bowtie2. Aligned reads were counted and summarized for the annotated genes using featureCounts. Differential gene expression analysis was performed by DESeq2. Differential expressed protein-coding genes with a log_2_ fold-change >0.6 or <−0.6 and adjusted *p*-value <0.05 were used for pathway analysis using DAVID.^56^

### Alkaline Phosphatase

*S. aureus* was grown to an OD_600_ of 0.2 (5 mL) and HACC CD was added within the range of 1000 to 0.2 μg/mL at pH 5.5 and 7.0 (15 mM H_2_O_2_ serving as the positive killing control) for 6 h. The solution was centrifuged at 10,000*g* for 10 min, and 1 mL of the supernatant was mixed with 2-amino 2-methyl 1-propanol buffer (pH 10.5) containing paranitrophenyl phosphate (PNPP) at a 2 mg/mL concentration. The samples were incubated at 30 °C for 30 min, and absorbance was measured using a quartz cuvette.

### ROS Generation with Ascorbic Acid

AA can be oxidized by ROS forming dehydroascorbic acid. Co-CQDs’ capacity to generate ROS at different pH’s was determined by measuring a decrease in absorbance from AA (absorption peak at 266 nm) when in the presence of Co-CQQDs. PBS buffer was adjusted to pH 5.5 and 7.0 with AA, and Co-CQDs were introduced at concentrations ranging from 12.5 to 100 μg/mL. The solutions were incubated for 2 h at 37 °C and ascorbic acid’s characteristic peak at OD_266_ was measured in a quartz cuvette. Above 100 μg/mL Co-CQD concentrations, the AA absorbance peak at OD_266_ was overshadowed by the absorbance of Co-CQD particles, and so, only low HACC CD concentrations were measured.

### Superoxide with Nucleic Acid

To investigate the capacity of Co-CQDs to generate superoxide radicals, dihydroethidium (DHE) was used as the fluorescent probe. Dihydroethidium was dissolved in DMSO and then resuspended in PBS to make a working solution of 10 μM. MRSA cells (1 mL, OD_600_ = 0.2) were subjected to Co-CQDs 1.2–150 μg/mL at 37 °C for 1 h. The cells were centrifuged, and the pellet was retained and incubated in DHE working solution for 30 min. The cells were then centrifuged, washed three times with PBS, and fluorescence was measured at λ_ex_/λ_em_ = 518 nm/606 nm.

### Membrane Polarization

MRSA, *E. coli*, and *E. faecalis* (25 μL, OD_600_ = 0.10) were added to 125 μL of 3,3′-dipropylthiadicarbocyanine iodide (diSC_3_-5) made to a concentration of 1.0 μM in HEPES buffer (10 mM) s supplemented with glucose, KCl, and MgSO_4_ at pH 5.5 and 7.0. The solution was allowed to equilibrate for 30 min before the addition of Co-CQDs (37.5–1000 μg/mL). The fluorescence was monitored at 2 h using a microplate reader (Tecan SPARK), λ_ex_/λ_em_ = 622 nm/674 nm.

### Hydrogen Peroxide Generation Assay (Amplex Red)

To determine the generation of hydrogen peroxide from Co-CQD nanoparticles, an Amplex Red assay was conducted as per the manufacturer’s instructions (Amplex Red Kit: Invitrogen A22188). Briefly, Co-CQD nanoparticles (2.3–300 μg/mL) were incubated with 100 μM Amplex Red reagent and 0.2 U/mL horseradish peroxidase for 30 min in the dark at room temperature. The positive control consisted of 15 mM H_2_O_2_, and the negative control was pure water. Fluorescence spectra indicative of H_2_O_2_ production were measured at λ_ex_/λ_em_ = 560 nm/580–650 nm.

### Live/Dead

Human dFIB (CC-2509-LONZA) were cultured in DMEM (10% FBS and 3% glutamine). Cells were grown to between 50 and 90% confluency in IBIDI 8-welled plates and Co-CQD nanoparticles were added at different concentrations (0, 10, 50, 100, and 500 μg/mL) and incubated for 24 h at pH 7 and 5.5 (acetic acid-adjusted). Hydrogen peroxide (15 mM) was the positive killing control. Cytotoxicity was determined by a LIVE/DEAD Cell Imaging assay (R37601-Invitrogen). Briefly, the kit reagents were added to cells as per the manufacturer’s suggestions, incubated for 20 min, and imaged on a Dragonfly 505 (Andor Technologies, Inc.) using 60× objective with oil immersion. Images were taken using excitation/emission wavelengths of 488/515 and 570/600 nm.

### Fibroblast Proliferation

Human dFIB (CC-2509-LONZA) cultured in DMEM (10% FBS and 3% glutamine) were grown to 80–90% confluency in Incucyte ImageLock 96-well plates (Sartorius) and a 700–800 μm scratch was made in cells using an Incucyte 96-Well Woundmaker. The cells were washed with PBS and then exposed to growth media at pH 7.0 and 5.5 (acetic acid-adjusted) containing Co-CQD nanoparticles at 500 μg/mL for 24 h. Following this, the medium was removed and replaced with fresh, unamended medium for a further 72 h. The cells were incubated and imaged in an IncuCyte Live-Cell Analysis System. Confluence was measured by the area of the scratch filled with migrating cells over time.

### Antimicrobial Evaluation in Mice

Mouse experiments were approved by the Norwegian Food and Animal Safety Authority (Mattilsynet) FOTS ethics approval no. 30052. All surgeries were performed under general anesthetic conditions to minimize suffering, and mice were randomly assigned for analysis.

The dorsal regions of female C57BL/6 mice (8–10 weeks) were shaved, and hair was further removed using Veet hair removal cream. A single-punch biopsy wound was made on each mouse (5 mm). The wounds were infected with MRSA (1.3 × 10^^7^ CFU) and allowed to dry for 3 h followed by the addition of Co-CQD nanoparticles in PBS at pH 5.5 (0.87 mg/kg ± 10%). The wounds were kept from contracting by gluing a splint around the wound. The wound was then covered by a commercial transparent wound dressing (Tegaderm Film, 3M). The mice (each treatment *n* = 6) were sacrificed after 24 h and their wounds were removed with scissors. Three wounds were homogenized and mixed in 1 mL of sterile PBS, and 10 μL of this was spread onto agar plates. The remaining 3 wounds were collected and paraffin-embedded for H&E staining and antibody labeling.

### Wound Healing Evaluation in Mice

The dorsal regions of Female C57BL/6 mice (8–10 weeks) were shaved and hair was further removed using Veet hair removal cream. A single excisional skin wound was made, as previously described. The wounds were infected with MRSA (2.2 × 10^6^ CFU) and allowed to dry for 3 h followed by the addition of Co-CQD nanoparticles in PBS at pH 5.5 (1.09 mg/kg ± 10%). The wounds were kept from contracting by gluing and suturing a silicone splint around the wound. The wound was then covered by a commercial transparent wound covering (Tegaderm Film, 3M). The wound covering was removed following 24 h and the splint was removed after 3 days (following scab formation). Mice were weighed every 1–2 days for 7 days and wound lengths and widths were measured with electronic calipers. The mice (each treatment *n* = 6) were sacrificed after 7 days, and their wounds were removed with scissors. Three wounds were homogenized and mixed in 10 mL of sterile PBS, and 10 μL of this was spread onto agar plates and incubated at 37 °C for 48 h. The remaining 3 wounds were collected and paraffin-embedded for H&E staining and antibody labeling.

### H&E Staining

Samples were fixed in formalin (10%), dehydrated through increasing ethanol steps (70–100%), and then cleared in xylene before paraffin infiltration. Samples were then embedded and sectioned. The HE (hematoxylin and eosin) staining included removing the paraffin (dewaxing), rehydration, staining with hematoxylin, rinsing, staining with eosin, rinsing, dehydration, clearing, and mounting. Samples were then imaged by using the Olympus VS120 slide scanner.

### Tissue Immunofluorescence

For tissue immunofluorescence, 5 mM tissue sections were deparaffinized in xylene (10 min twice) and rehydrated by immersing in a series of graded alcohols (100% EtOH 10 min twice, 95% EtOH for 10 min once, 70% EtOH for 10 min twice) and distilled water (5 min twice). Antigen retrieval was performed by boiling the sections in a pressure cooker at 98 °C for 12 min. For staining of MPO and iNOS, antigen retrieval was performed using Tris-based antigen unmasking solution, pH 9.0 (Vector Laboratories, H-3301) whereas antigen unmasking for F4/80 was performed in sodium citrate buffer, pH 6.0 (Vector Laboratories, H-3300). After cooling at RT for 40 min, sections for F4/80 were blocked in 10% normal goat serum (Invitrogen, 50197Z), whereas 5% bovine serum albumin diluted in PBS was used for MPO and iNOS. Following blocking for 1 h at room temperature, slides were incubated at 4 °C overnight with primary antibodies. F4/80 (Cell Signaling, 70076) and MPO (R&D systems, AF3667) were diluted 1:200, whereas iNOS (Abcam, ab15323) was diluted 1:100, respectively. After washing in PBS containing 0.05% Tween-20, samples were incubated for 1 h in the dark with secondary antibody, diluted 1:200 (Alexa-Fluor 647: Invitrogen, A21244, Alexa-Fluor 546: Invitrogen, A11056). After the final washing, sections were mounted using Prolong Diamond antifade reagent containing DAPI (Invitrogen, P36970). Images were acquired using an Olympus VS120 slide scanning system (40× objective).

### Trichrome Stain

Collagenous connective tissue fibers were visualized using the Trichrome Stain kit from Abcam (ab150686). In brief, 5 mM sections were deparaffinized and rehydrated, as described under the tissue immunofluorescent section. Next, slides were stained according to the manufacturer’s instructions. Slides were cleared in xylene and mounted with Eukitt Quick-hardening mounting media (Sigma-Aldrich, 03989). Images were obtained on an Olympus VS120 slide scanning system (40× objective).

### Statistics and Reproducibility

All microscopy including TEM and confocal images were independently repeated in triplicate. Error bars are representative of standard deviation from the mean. Mice were randomly assigned to treatment groups and randomly assigned to wound bacterial load or wound histology investigations. Statistical analysis between treatment groups were determined by one-way or two-way analysis of variances, complimented by Tukey’s post hoc analysis. In certain assays, unpaired *t*-test with Welch correction was employed. All statistical analysis used a 95% confidence interval (*p*-value <0.05).

## Supplementary Material



## Notes

TEM of Co-CQD; Co-CQD characterization; antibacterial activity of Co-CQDs; Co-CQD treatment *in vivo*; and mechanism of Co-CQDs’ antibacterial activity (TEM of Co-CQD, Co-CQD characterization, antibacterial activity of Co-CQDs, Co-CQD treatment removes infection *in vivo*, mechanisms of Co-CQDs antibacterial activity) (PDF)

## References

[1] WHO . 2023 Antibacterial Agents in Clinical and Preclinical Development: An Overview and Analysis; World Health Organization: Geneva, 2024; Licence: CC BY-NC-SA 3.0 IGO.

[2] Frost I., Sati H., Garcia-Vello P., Hasso-Agopsowicz M., Lienhardt C., Gigante V., Beyer P. (2023). The role of
bacterial
vaccines in the fight against antimicrobial resistance: an analysis
of the preclinical and clinical development pipeline. Lancet Microbe.

[3] Epple M., Rotello V. M., Dawson K. (2023). The Why and How of Ultrasmall Nanoparticles. Acc. Chem. Res..

[4] Geng B., Hu J., Li Y., Feng S., Pan D., Feng L., Shen L. (2022). Near-infrared
phosphorescent carbon dots for sonodynamic precision
tumor therapy. Nat. Commun..

[5] Li S., Su W., Wu H., Yuan T., Yuan C., Liu J., Deng G., Gao X., Chen Z., Bao Y., Yuan F., Zhou S., Tan H., Li Y., Li X., Fan L., Zhu J., Chen A. T., Liu F., Zhou Y., Li M., Zhai X., Zhou J. (2020). Targeted tumour
theranostics in mice via carbon quantum dots structurally mimicking
large amino acids. Nat. Biomed. Eng..

[6] Zhang T., Wang B., Cheng Q., Wang Q., Zhou Q., Li L., Qu S., Sun H., Deng C., Tang Z. (2024). Polaron engineering
promotes NIR-II absorption of carbon quantum dots for bioimaging and
cancer therapy. Sci. Adv..

[7] Kumar V. B., Sher I., Rencus-Lazar S., Rotenstreich Y., Gazit E. (2023). Functional Carbon Quantum Dots for Ocular Imaging and Therapeutic
Applications. Small.

[8] Cui T., Yu J., Wang C. F., Chen S., Li Q., Guo K., Qing R., Wang G., Ren J. (2022). Micro-Gel Ensembles
for Accelerated Healing of Chronic Wound via pH Regulation. Adv. Sci..

[9] Schultz, G. S. ; Chin, G. A. ; Moldawer, L. ; Diegelmann, R. F. In Mechanisms of Vascular Disease: A Reference Book for Vascular Specialists; Fitridge, R. , Thompson, M. , Eds.; University of Adelaide Press: Adelaide (AU), 2011. 30484990

[10] Monack D.
M., Mueller A., Falkow S. (2004). Persistent bacterial infections:
the interface of the pathogen and the host immune system. Nat. Rev. Microbiol..

[11] Cui T., Yu J., Wang C. F., Chen S., Li Q., Guo K., Qing R., Wang G., Ren J. (2022). Micro-Gel Ensembles
for Accelerated Healing of Chronic Wound via pH Regulation. Adv. Sci..

[12] Guan Y., Niu H., Liu Z., Dang Y., Shen J., Zayed M., Ma L., Guan J. (2021). Sustained oxygenation accelerates diabetic wound healing
by promoting epithelialization and angiogenesis and decreasing inflammation. Sci. Adv..

[13] Dargaville T. R., Farrugia B. L., Broadbent J. A., Pace S., Upton Z., Voelcker N. H. (2013). Sensors and imaging for wound healing: A review. Biosens. Bioelectron..

[14] Percival S. L., McCarty S., Hunt J. A., Woods E. J. (2014). The effects of pH
on wound healing, biofilms, and antimicrobial efficacy. Wound Repair Regen..

[15] Kuo S.-H., Shen C.-J., Shen C.-F., Cheng C.-M. (2020). Role of pH Value
in Clinically Relevant Diagnosis. Diagnostics.

[16] Agrawal K. S., Sarda A. V., Shrotriya R., Bachhav R., Puri V., Nataraj G. (2017). Acetic acid dressings:
Finding the Holy Grail for infected
wound management. Indian J. Plast. Surg..

[17] Ryssel H., Kloeters O., Germann G., Schäfer T., Wiedemann G., Oehlbauer M. (2009). The antimicrobial effect of acetic
acid—An alternative to common local antiseptics?. Burns.

[18] Feng L., Xu M., Zeng W., Zhang X., Wang S., Yao Z., Zhou T., Shi S., Cao J., Chen L. (2022). Evaluation
of the antibacterial, antibiofilm, and anti-virulence effects of acetic
acid and the related mechanisms on colistin-resistant Pseudomonas
aeruginosa. BMC Microbiol..

[19] Phillips I., Lobo A. Z., Fernandes R., Gundara N. S. (1968). Acetic acid in the
treatment of superficial wounds infected by Pseudomonas aeruginosa. Lancet.

[20] Leveen H. H., Falk G., Borek B., Diaz C., Lynfield Y., Wynkoop B. J., Mabunda G. A., Rubricius J. L., Christoudias G. C. (1973). Chemical acidification of wounds.
An adjuvant to healing
and the unfavorable action of alkalinity and ammonia. Ann. Surg..

[21] Sun W., Feng L., Zhang J., Lin K., Wang H., Yan B., Feng T., Cao M., Liu T., Yuan Y., Wang N. (2022). Amidoxime Group-Anchored Single Cobalt
Atoms for Anti-Biofouling
during Uranium Extraction from Seawater. Adv.
Sci..

[22] Gao Y. G., Sriram M., Wang A. H. (1993). Crystallographic
studies of metal
ion-DNA interactions: different binding modes of cobalt­(II), copper­(II)
and barium­(II) to N7 of guanines in Z-DNA and a drug-DNA complex. Nucleic Acids Res..

[23] Kou X., Jiang S., Park S. J., Meng L. Y. (2020). A review: recent
advances in preparations and applications of heteroatom-doped carbon
quantum dots. Dalton Trans..

[24] Truskewycz A., Yin H., Halberg N., Lai D. T., Ball A. S., Truong V. K., Rybicka A. M., Cole I. (2022). Carbon Dot Therapeutic Platforms:
Administration, Distribution, Metabolism, Excretion, Toxicity, and
Therapeutic Potential. Small.

[25] Papadopoulou V., Sidders A., Lu K., Velez A., Durham P., Bui D., Angeles-Solano M., Dayton P., Rowe S. (2023). Overcoming
biological barriers to improve treatment of a Staphylococcus aureus
wound infection. Cell Chem. Biol..

[26] Kharaziha M., Baidya A., Annabi N. (2021). Rational Design
of Immunomodulatory
Hydrogels for Chronic Wound Healing. Adv. Mater..

[27] da
Silva R. A. G., Wong J. J., Antypas H., Choo P. Y., Goh K., Jolly S., Liang C., Tay Kwan Sing L., Veleba M., Hu G., Chen J., Kline K. (2023). Mitoxantrone
targets both host and bacteria to overcome vancomycin resistance in
Enterococcus faecalis. Sci. Adv..

[28] Hirshfield I. N., Terzulli S., O’Byrne C. (2003). Weak organic acids: a panoply of
effects on bacteria. Sci. Prog..

[29] Zhou C., Fey P. D. (2020). The acid response network of Staphylococcus
aureus. Curr. Opin. Microbiol..

[30] Madhusudhan V. L. (2016). Efficacy
of 1% acetic acid in the treatment of chronic wounds infected with
Pseudomonas aeruginosa: prospective randomised controlled clinical
trial. Int. Wound J..

[31] Meng L., Liu S., Borsa B. A., Eriksson M., Mak W. C. (2024). A conducting polymer-based
array with multiplex sensing and drug delivery capabilities for smart
bandages. Commun. Mater..

[32] Thoma-Uszynski S., Stenger S., Takeuchi O., Ochoa M. T., Engele M., Sieling P. A., Barnes P. F., Röllinghoff M., Bölcskei P. L., Wagner M., Akira S., Norgard M. V., Belisle J. T., Godowski P. J., Bloom B. R., Modlin R. L. (2001). Induction
of Direct Antimicrobial Activity Through Mammalian Toll-Like Receptors. Science.

[33] Tezuka H., Abe Y., Iwata M., Takeuchi H., Ishikawa H., Matsushita M., Shiohara T., Akira S., Ohteki T. (2007). Regulation of IgA production
by naturally occurring TNF/iNOS-producing dendritic cells. Nature.

[34] Sweet M. J., Ramnath D., Singhal A., Kapetanovic R. (2025). Inducible
antibacterial responses in macrophages. Nat.
Rev. Immunol..

[35] Kim J., Liu L., Vázquez-Torres A. (2021). The DnaK/DnaJ
Chaperone System Enables
RNA Polymerase-DksA Complex Formation in Salmonella Experiencing Oxidative
Stress. mBio.

[36] Sun D., Liu Y., Peng X., Dong H., Jiang H., Fan X., Feng Y., Sun J., Han K., Gao Q., Niu J., Ding J. (2023). ClpP protease modulates bacterial growth, stress response,
and bacterial virulence in Brucella abortus. Vet. Res..

[37] Fritsch V. N., Loi V. V., Busche T., Tung Q. N., Lill R., Horvatek P., Wolz C., Kalinowski J., Antelmann H. (2020). The alarmone (p)­ppGpp confers tolerance
to oxidative
stress during the stationary phase by maintenance of redox and iron
homeostasis in Staphylococcus aureus. Free Radical
Biol. Med..

[38] Talbott H. E., Mascharak S., Griffin M., Wan D. C., Longaker M. T. (2022). Wound healing,
fibroblast heterogeneity, and fibrosis. Cell
Stem Cell.

[39] Yokota M., Häffner N., Kassier M., Brunner M., Shambat S. M., Brennecke F., Schniering J., Marques Maggio E., Distler O., Zinkernagel A. S., Maurer B. (2021). Staphylococcus aureus
impairs dermal fibroblast functions with deleterious effects on wound
healing. FASEB J..

[40] Shaw Z. L., Kuriakose S., Cheeseman S., Dickey M. D., Genzer J., Christofferson A. J., Crawford R. J., McConville C. F., Chapman J., Truong V. K., Elbourne A., Walia S. (2021). Antipathogenic
properties and applications of low-dimensional materials. Nat. Commun..

[41] Wang Y., Shi H., Zhang H., Yu Chen Y., Ren B., Tang Q., Sun Q., Zhang Q., Liu J. (2023). A Multifunctional Nanozyme with NADH
Dehydrogenase-Like Activity and Nitric Oxide Release under Near-Infrared
Light Irradiation as an Efficient Therapeutic for Antimicrobial Resistance
Infection and Wound Healing. Adv. Healthcare
Mater..

[42] Li B., Luo Y., Zheng Y., Liu X., Tan L., Wu S. (2022). Two-dimensional
antibacterial materials. Prog. Mater. Sci..

[43] Sun W., Wu F.-G. (2018). Two-Dimensional Materials for Antimicrobial Applications:
Graphene
Materials and Beyond. Chem.—Asian J..

[44] Zhang Z., Qian L., Zhang N., Wang X., Fu Y., Gao G., Sun T. (2025). Advances in Spiky Antibacterial Materials:
From Bioinspired
Design to Application. Small Struct..

[45] He S., Huang J., Zhang Q., Zhao W., Xu Z., Zhang W. (2021). Bamboo-Like Nanozyme Based on Nitrogen-Doped Carbon Nanotubes Encapsulating
Cobalt Nanoparticles for Wound Antibacterial Applications. Adv. Funct. Mater..

[46] Perreault F., de Faria A. F., Nejati S., Elimelech M. (2015). Antimicrobial
Properties of Graphene Oxide Nanosheets: Why Size Matters. ACS Nano.

[47] Li J., Zhang M., Wang Y., Lv W., Xu Z., Wang B., Huang R., Mei B., Wang Y. (2024). Regulating
the Atomic Active Center by Covalent Organic Framework-Derived Photothermal
Nanozyme to Arm Self-Gelling Powder for Bacterial Wound Healing. ACS Nano.

[48] Nagoba B. S., Selkar S. P., Wadher B. J., Gandhi R. C. (2013). Acetic acid treatment
of pseudomonal wound infections – A review. J. Infect. Public Health.

[49] Surwade P., Ghildyal C., Weikel C., Luxton T., Peloquin D., Fan X., Shah V. (2019). Augmented antibacterial
activity of ampicillin with
silver nanoparticles against methicillin-resistant Staphylococcus
aureus (MRSA). J. Antibiot..

[50] Ayala-Núñez N. V., Lara Villegas H. H., del Carmen Ixtepan Turrent L., Rodríguez
Padilla C. (2009). Silver Nanoparticles Toxicity and Bactericidal Effect
Against Methicillin-Resistant Staphylococcus aureus: Nanoscale Does
Matter. NanoBiotechnology.

[51] Zhang Q., Deng S., Liu J., Zhong X., He J., Chen X., Feng B., Chen Y., Ostrikov K. (2019). Cancer-Targeting
Graphene Quantum Dots: Fluorescence Quantum Yields, Stability, and
Cell Selectivity. Adv. Funct. Mater..

[52] Liu J., Yu M., Zhou C., Zheng J. (2013). Renal clearable inorganic nanoparticles:
a new frontier of bionanotechnology. Mater.
Today.

[53] Zomer H. D., Trentin A. G. (2018). Skin wound healing
in humans and mice: Challenges in
translational research. J. Dermatol. Sci..

[54] Falcone M., De Angelis B., Pea F., Scalise A., Stefani S., Tasinato R., Zanetti O., Dalla Paola L. (2021). Challenges
in the management of chronic wound infections. J. Glob. Antimicrob. Resist..

[55] Chen P.-J., Hua Y.-M., Toh H. S., Lee M.-C. (2022). Topical antibiotic
prophylaxis for surgical wound infections in clean and clean-contaminated
surgery: a systematic review and meta-analysis. BJS Open.

[56] Sherman B. T., Hao M., Qiu J., Jiao X., Baseler M. W., Lane H. C., Imamichi T., Chang W. (2022). DAVID: a web
server for functional
enrichment analysis and functional annotation of gene lists (2021
update). Nucleic Acids Res..

