# Supplementary material for: Cobalt-Doped Carbon Quantum Dots Work Synergistically with Weak Acetic Acid to Eliminate Antimicrobial-Resistant Bacterial Infections
Source: ACS Nano. 2025 Sep 8;19(37):33103–17. doi: 10.1021/acsnano.5c03108 (PMC12462238; doi:10.1021/acsnano.5c03108)
Supplement: Supplementary file 1 [file nn5c03108_si_001.pdf]

Supplementary Materials for

Cobalt doped carbon quantum dots work synergistically with weak acetic acid to eliminate antimicrobial resistant bacterial infections

*Adam Truskewycz[1, 2, *], Benedict Choi[1,3,4,5,6], Line Pedersen[1], Jianhua Han[1], Melanie MacGregor[2], Nils Halberg[1,7, *]*

[1] Department of Biomedicine, University of Bergen, Bergen, Norway, 5009

[2] Flinders Institute for Nanoscale Science and Technology, College of Science and Engineering, Flinders University, South Australia, Australia, 5042

[3] Department of Biochemistry and Biophysics, University of California, San Francisco, CA, USA, 94143

[4] Department of Urology, University of California, San Francisco, CA, USA, 94143

[5] Helen Diller Family Comprehensive Cancer Center, University of California, San Francisco, CA, USA, 94143

[6] Bakar Computational Health Sciences Institute, University of California, San Francisco, CA, USA, 94143

[7] Cancer Research Program, QIMR Berghofer Medical Research Institute, Brisbane, Australia, 4006

*Corresponding author. Email: adam.trusk@gmail.com, nils.halberg@qimrberghofer.edu.au

Fig. S1.

a

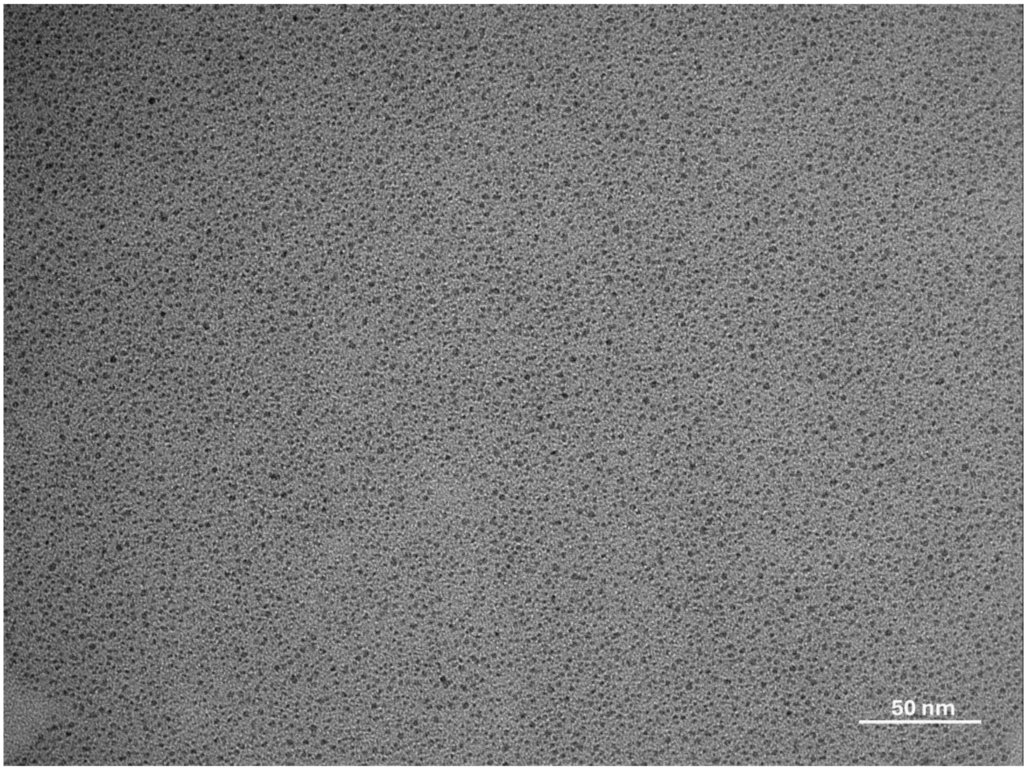

b

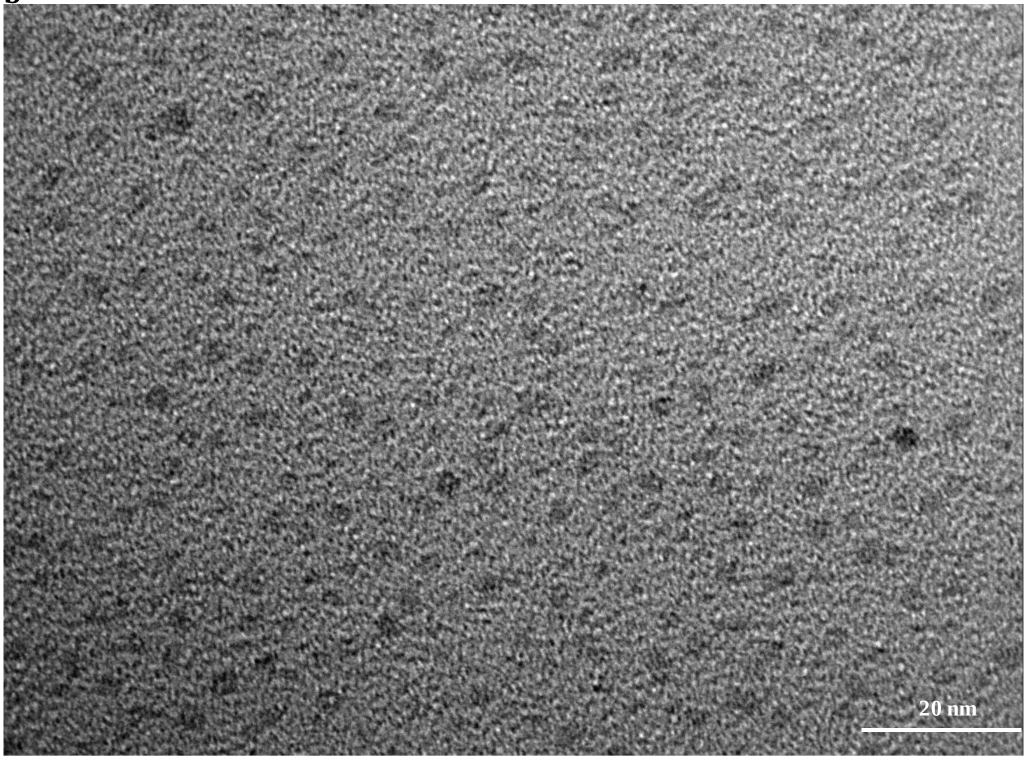

TEM of Co-CQD: Transmission electron micrograph of Co-CQD nanoparticles at **a**, 150,000 X magnification and **b**, 600,000 X magnification

Fig. S2.

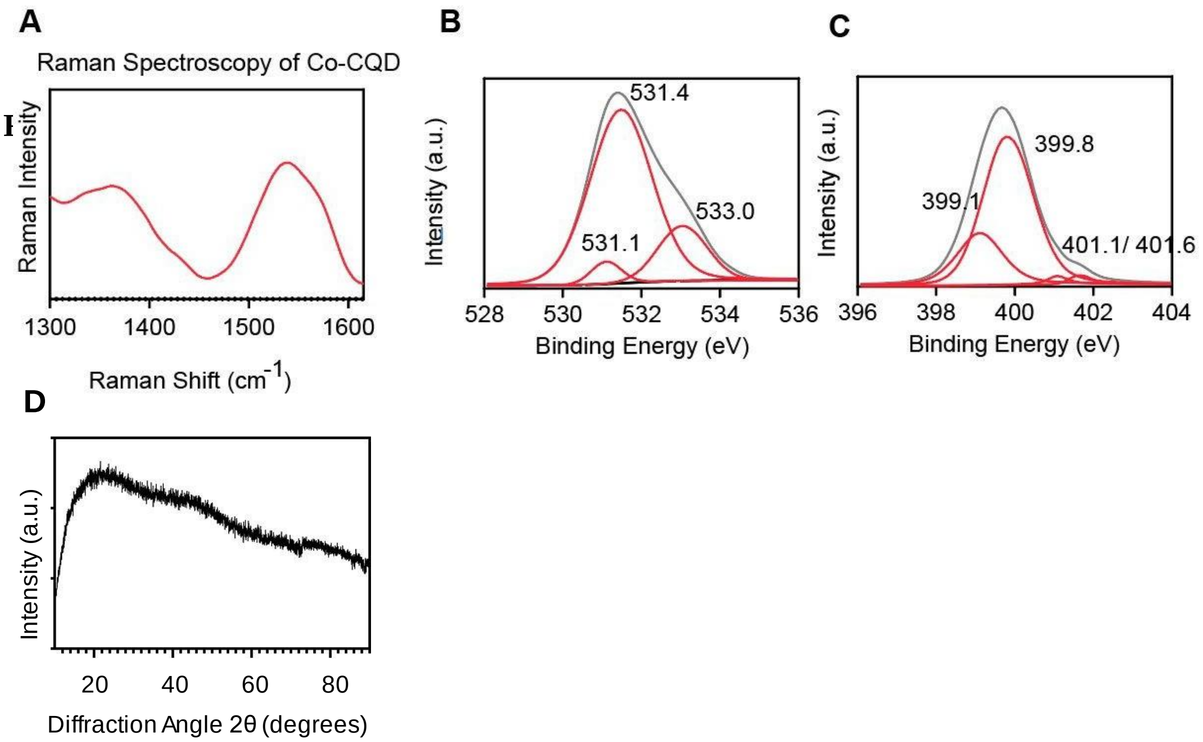

Co-CQD characterisation: **a**, Raman spectra of Co-CQDs between 13001600 cm^{-1}. **b**, X-ray photoelectron spectroscopy (XPS) of O1s and **c,** N1s peaks from Co-CQDs. **d**, X-ray diffraction

Fig. S3.

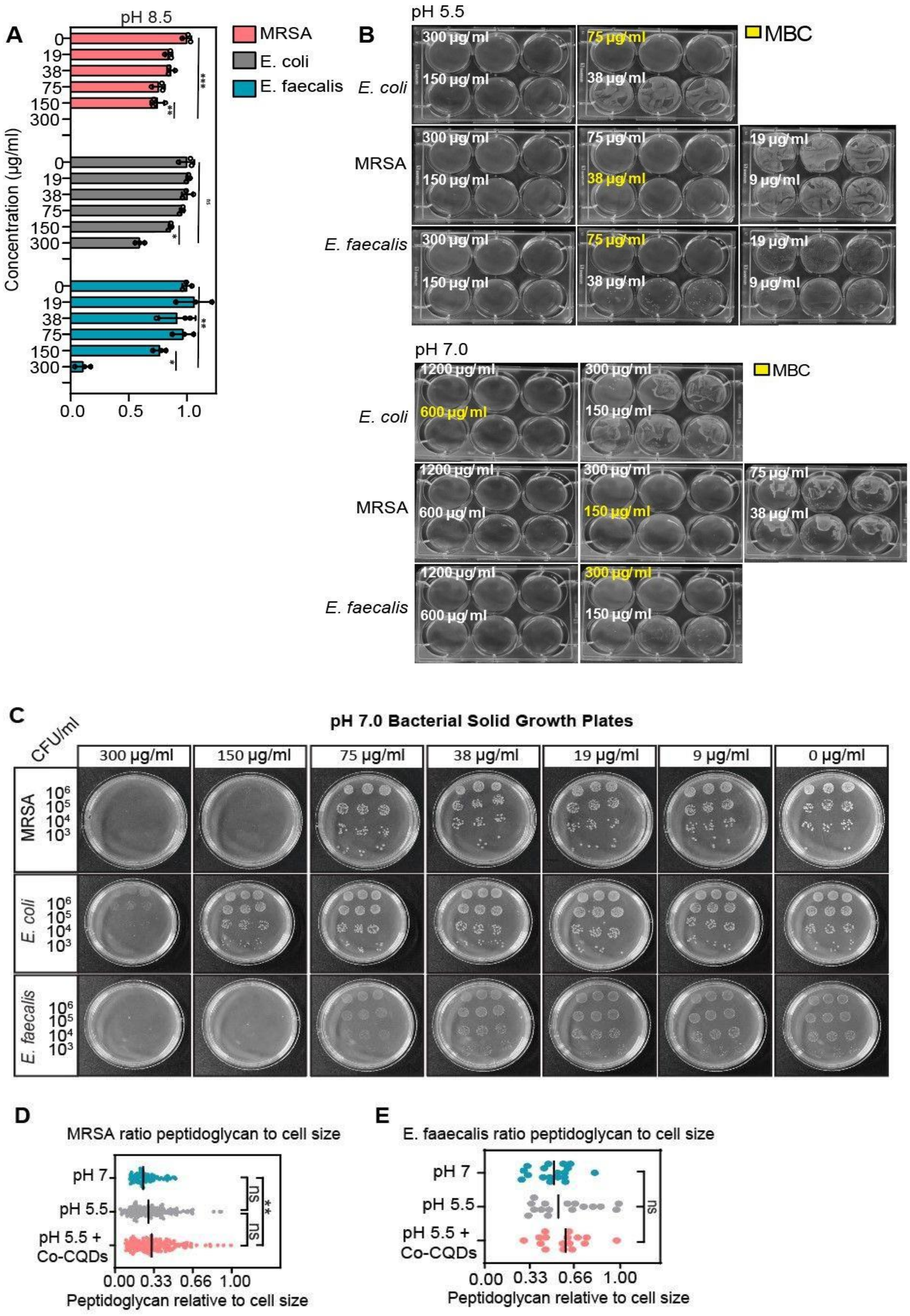

A
pH 8.5
MRSA
E. coli
E. faecalis
Concentration (μg/ml)
0
19
38
75
150
300
0.0
0.5
1.0
B
pH 5.5
MBC
E. coli
MRSA
E. faecalis
300 μg/ml
150 μg/ml
75 μg/ml
38 μg/ml
19 μg/ml
9 μg/ml
pH 7.0
1200 μg/ml
600 μg/ml
C
pH 7.0 Bacterial Solid Growth Plates
CFU/ml
300 μg/ml
150 μg/ml
75 μg/ml
38 μg/ml
19 μg/ml
9 μg/ml
0 μg/ml
10^6
10^5
10^4
10^3
D
MRSA ratio peptidoglycan to cell size
pH 7
pH 5.5
pH 5.5 +
Co-CQDs
0.00 0.33 0.66 1.00
Peptidoglycan relative to cell size
E
E. faaecalis ratio peptidoglycan to cell size

Antibacterial activity of Co-CQDs. **a**, Optical density ($OD_{600\ nm}$) of MRSA, *E. coli,* and *E. faecalis* in pH adjusted liquid growth cultures after 24 h exposure to Co-CQDs at pH 8.5. **b**, MBC of MRSA, *E. coli,* and *E. faecalis* from the aqueous growth assay supplemented with differing Co-CQD concentrations at pH 5.5 (0 – 300 µg/ml) and pH 7.0 (0 – 1,200 µg/ml). **c**, Growth of MRSA, *E. coli*, and *E. faecalis* on nutrient agar plates supplemented with differing CoCQD concentrations (0 – 300 µg/ml) at pH 7.0. Each plate has been seeded with different concentrations of bacteria (5.1 X 10^6, 10^5, 10^4, and 10^3 CFU/ ml) from top to bottom in triplicate, **d**, Ratio of *MRSA* peptidoglycan content relative to cell size **e**, Ratio of *E. faecalis* peptidoglycan content relative to cell size

Fig. S4.

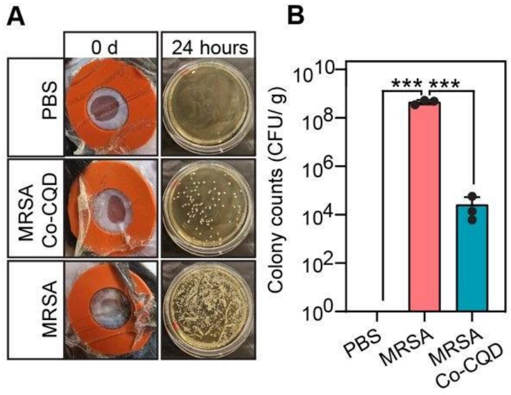

Co-CQD treatment removes infection *in vivo*. a, Wound areas following 24 h incubation with PBS, Co-CQD + MRSA and MRSA with agar plates representing their wound bacterial load after 24 h exposure. b, quantification of bacterial presence in wound areas following treatments after 24h. Statistical significance was determined using a one-way analysis of variance (ANOVA) with Tukey's multiple comparison test. ns, *, **, ***, **** signifies not significant, $p < 0.05$, $p < 0.005$, $p < 0.0005$ and $p < 0.0001$, respectively.

Fig. S5.

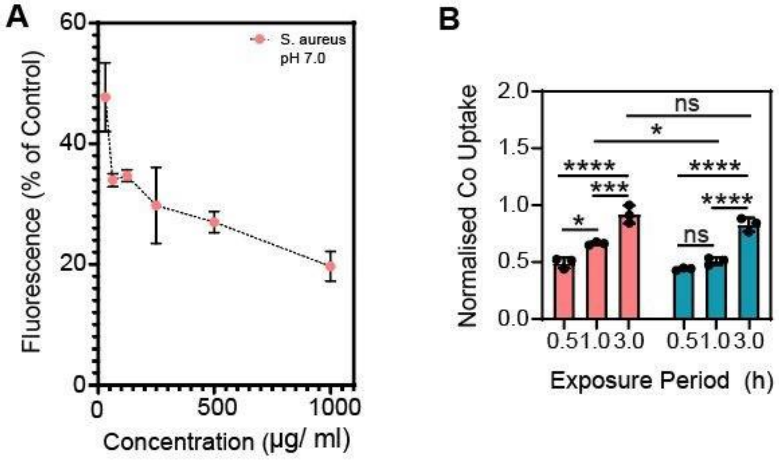

Mechanisms of Co-CQDs antibacterial activity. a, Membrane hyperpolarisation of MRSA at pH 7.0 resulting from differing Co-CQD exposure. b, Normalised uptake of Co-CQD at pH 5.5 and 7.0 measured through cobalt concentration at 0.5, 1, and 3 h time periods. Statistical significance was determined using (a) Unpaired t test with Welch correction, and (b) a one-way analysis of variance (ANOVA) with Tukey's multiple comparison test. ns, *, **, ***, **** signifies not significant, $p < 0.05$, $p < 0.005$, $p < 0.0005$ and $p < 0.0001$, respectively.
